# Draft genome assembly of *Tenualosa ilisha*, Hilsa shad, provides resource for osmoregulation studies

**DOI:** 10.1038/s41598-019-52603-w

**Published:** 2019-11-11

**Authors:** Vindhya Mohindra, Tanushree Dangi, Ratnesh K. Tripathi, Rajesh Kumar, Rajeev K. Singh, J. K. Jena, T. Mohapatra

**Affiliations:** 1 ICAR-National Bureau of Fish Genetic Resources (NBFGR), Canal Ring Road, P.O. Dilkusha, Lucknow, 226 002 India; 2 Indian Council of Agricultural Research (ICAR), Krishi Anusandhan Bhawan - II, New Delhi, 110 012 India; 3 Present Address: Imperial Life Sciences (P) Limited, Gurgaon, Haryana 122001 India

**Keywords:** Conservation genomics, DNA metabolism

## Abstract

This study provides the first high-quality draft genome assembly (762.5 Mb) of *Tenualosa ilisha* that is highly contiguous and nearly complete. We observed a total of 2,864 contigs, with 96.4% completeness with N_50_ of 2.65 Mbp and the largest contig length of 17.4 Mbp, along with a complete mitochondrial genome of 16,745 bases. A total number of 33,042 protein coding genes were predicted, among these, 512 genes were classified under 61 Gene Ontology (GO) terms, associated with various homeostasis processes. Highest number of genes belongs to cellular calcium ion homeostasis, followed by tissue homeostasis. A total of 97 genes were identified, with 16 GO terms related to water homeostasis. Claudins, Aquaporins, Connexins/Gap junctions, Adenylate cyclase, Solute carriers and Voltage gated potassium channel genes were observed to be higher in number in *T. ilisha*, as compared to that in other teleost species. Seven novel gene variants, in addition to claudin gene (CLDZ), were found in *T. ilisha. The* present study also identified two putative novel genes, NKAIN3 and L4AM1, for the first time in fish, for which further studies are required for pinpointing their functions in fish. In addition, 1.6 million simple sequence repeats were mined from draft genome assembly. The study provides a valuable genomic resource for the anadromous Hilsa. It will form a basis for future studies, pertaining to its adaptation mechanisms to different salinity levels during migration, which in turn would facilitate in its domestication.

## Introduction

*Tenualosa ilisha* (Hamilton, 1822), commonly known as Hilsa shad, belongs to Clupeidae family. It is an anadromous fish, with wide distribution in Southeast and South Asia^1,2^, ranging from China Sea, Bay of Bengal, Arabian Sea, Red Sea to Persian Gulf and is also found in coastal areas, estuaries and freshwater rivers^3^. It is commercially important due to its higher content of omega fatty acid and potential for therapeutic applications^4^. A recent review^4^ highlighted the biology, migration, ecology and genetics of Hilsa with respect to its future sustainability, however, mechanism underlying adaptation to varying salinity levels during its migration, is still not understood.

Hilsa usually inhabits coastal and estuarine waters, with varying levels of salinity (22.4–33.4 ppt)^5,6^. During its spawning migration, Hilsa ascends to freshwater with low salinity (approximately 0.05 ppt)^5^. However, for growth as well as for feeding, young ones migrate towards the sea.Thus, during its life cycle, Hilsa experiences large salinity level variations and exhibits significant physiological adaptations between hyper-osmotic seawater and hypo-osmotic freshwater environments.

Declines in production of Hilsa from natural population have raised concerns and it may probably due to over exploitation and/or change in habitat^7,8^. There is an urgent need for development of technology for captive breeding, seed production and culture in confined water environment. Due to its anadromous nature, the captive breeding and farm rearing has long been regarded as a difficult task. In this direction, larval rearing in freshwater has been attempted for mass production of fry to support future programmes in Hilsa aquaculture^3^. To understand the hatching and rearing of larvae under captive conditions, it is necessary to study underlying molecular mechanisms of adaptation to salinity fluctuations during migration for spawning and growth, in Hilsa. Thus, development of the genomic resources, which forms a basis to determine genes responsible for homeostasis and osmoregulation, becomes necessary.

During the migration, from sea to freshwater and *vice versa*, the anadromous fish faces challenges in osmo-regulation processes, which majorly influences the ionic homeostasis. In such a situation, a fish osmoregulatory mechanisms cope with these changes by switching/regulating ion absorption (hyper-osmoregulation) and excretion (hypo-osmoregulation)^9^. For the purpose, several classes of ion transporters, pumps and channels are responsible for regulating ion uptake and secretion processes^10^. Historical genomic events such as teleost-specific whole-genome duplication and subsequent gene-family expansions have been reported to exhibit adaptability to variations in salinity^11^. A variety of gene families, claudins, aquaporins and ion channels^12,13^, have been reported to be associated with the salinity adaptations in fishes. In migratory fishes, the claudins, which shape the tight junctions, are usually present in high copy numbers, which can be due to gene duplication event independent of whole-genome duplication in teleost fish^14^. Their high copy number may relate to the modulation of activities required to adjust to dramatic changes, controlling tight junctions during fish adaptation to varying osmotic and ionic gradients^15^. Earlier findings also indicated the role of gap junctions/connexin proteins in osmoregulation, where the abundance of alpha and beta forms of gap junction proteins were observed in the gills of freshwater and saltwater fish, respectively^16^. In addition, in fish gills, the adenylate cyclase activity was found to be important for salt adaptation^17^.

In this direction, the genomic data of Hilsa, in the form of a high quality reference genome sequence is essential, which would provide a unique resource for comparative genomics for identifying molecular mechanism related to salinity adaptation. The genomic architecture will also enable to identify novel candidate genes and pathways responsible for salinity tolerance, which would in turn be a useful resource for assisting in the captive breeding and culture of Hilsa.

In this study, we report a high-quality genome of Hilsa, assembled using data generated from long read sequencing platform and error correction using short read sequences. The present assembly of Hilsa genome spans 762.5 Mb (∼96.4% of estimated genome size) and is highly contiguous with a N_50_ of 2.6 Mb and largest contig length of 17.4 Mbp. We identified 33,042 protein coding genes, which represent 95.8% of the euchromatin content. From this genome assembly, the various classes of homeostasis genes were identified and through comparative genomics, species specific as well as novel genes in Hilsa were also discovered.

## Materials and Methods

### Sample collection

Adult *T. ilisha* were collected from commercial catches, from natural freshwater habitat (Padama River; N 24° 80′, E 87° 93′, Farrrakka, West Bengal, India) and euthanized with MS222 (Sigma Aldrich, USA). Tissue samples of brain, liver, gill, testes and ovary were snap frozen in liquid N_2_, transported to the laboratory and stored at −80 °C, until analysis.

All the protocols followed were approved by Institute Animal Ethics Committee (IAEC), ICAR-NBFGR, vide No. G/CPCSEA/IAEC/2015/2 dated 27 Oct., 2015. All the methods were performed in accordance with the relevant guidelines and regulations.

### Genome sequencing

Genomic DNA (gDNA) was isolated from snap frozen testes tissue *via* phenol-chloroform method and purified with AMPure PB beads (Beckman Coulter, Inc., CA). For long read DNA sequencing, genomic SMRT bell libraries (10 and 20 Kbp size) were prepared from purified high-quality gDNA (10 µg) (from a single specimen) using the PacBio DNA template preparation kit 1.0 (Pacific Biosciences, Inc., Menlo Park, CA, USA) and were sequenced using P4-C2 and P6-C4 chemistry using PacBio RSII platform (Pacific Biosciences, Inc., CA). For short read genome sequencing, a total of three short-read genomic libraries, using the DNA sample prep kit, TruSeq (Illumina, San Diego, CA), were prepared from the same individual, as for PacBio sequencing and were used in paired-end (PE) sequencing (2 × 100 bp) on the Illumina HiSeq. 2500 sequencer (Illumina, CA). *T. ilisha* genome size was estimated by k-mer (k = 20) analysis with Jellyfish^18^ (version 2.2.3) using trimmed short read sequencing data. The counted K-mer was summarized as histogram and frequency distribution curve was plotted using R package^19^ (version R 3.5.1). Genome size was estimated as total number of k-mers/peak value of k-mer frequency distribution.

### Transcriptome (Iso-Seq) sequencing

Total RNA was isolated from five tissues, brain, gill, liver, testes and ovary samples, with NucleoSpin RNAII kit (Macherey-Nagel GmbH & Co. KG) following manufacturer recommendation. First strand cDNA synthesized were used to prepare SMRT bell libraries, using DNA template preparation kit 1.0 and sequenced with P6-C4 chemistry on the PacBio RSII platform. The raw reads generated were processed by RS_IsoSeq pipeline in Pacific Biosciences SMRT analysis software v2.3.0^20^ to classify full-length and non-full-length isoforms. All the full-length reads obtained from the same transcript isoform were clustered with Iso-Seq cluster algorithm, using a minimum (>=0.99) Quiver Accuracy^21^. Thereafter, the resulting consensus sequences were polished with non-full-length reads using the Quiver algorithm^21^.

### *De-novo* assembly of *T. ilisha* genome and statistics

The genome of *T. ilisha* was assembled from PacBio long reads using FALCON v.0.3.0, the diploid assembly approach^22^. Five assembly runs were compared for the mapping of seed reads over pre-assembly with length cut-off values in FALCON ranging from 6,000 to 10,000 bp. Based on the contig N_50_ results, length_cut-off = 8,000 was selected for the pre-assembly step. The assembled draft genome was self-corrected (using all raw PacBio generated reads) by Quiver tool^21^ and was further evaluated by mapping of Illumina reads in CLC genomic workbench 9.5.3^23^. The first round error corrected draft assembly was further error-corrected using short reads generated by Illumina platform by PILON^24^ tool. The final draft assembly was screened for any vector contamination using VecScreen software^25^. The mitochondrial sequences were separated out after BLAST searches against databases of mitochondrial sequences^26^. The final statistics of draft assembly was generated through QUAST^27^ with default parameters and was evaluated for its completeness using BUSCO v.3.0^28^ against the Actinopterygii (actinopterygii_odb9), Vertebrate (vertebrate_odb9) and Eukaryota (eukaryota_odb9) databases, containing a total of 4584, 2586 and 303 ortholog groups, respectively.

### Characterization of repetitive elements

The assembled draft genome (primary contigs) was characterized for repetitive elements using RepeatScout^29^. Mono to hexa-nucleotide simple sequence repeats (SSR) in the assembled genome were identified with the Microsatellite Identification tool MISA^30^; on default parameters. Transposable elements and miniature inverted-repeat were identified by TransposonPSI^31^ and TEclass^32^. Super families of transposable elements were predicted through MITE Digger^33^.

### Gene prediction and annotation of nuclear genome

The assembled draft genome was masked for highly repetitive DNA sequences and low-complexity regions using the Windowmasker programme^34^ and used for *de novo* gene prediction with Augustus^35^ (version 3.2.3). A total of three *de novo* prediction sets were generated by Augustus as follows: The first set of genes was predicted at default parameters using–species = zebrafish as the model species. For the second set, Augustus was trained to use *T. ilisha* as the species using the evidence of complete genes from earlier BUSCO results, followed by gene prediction with–species = *T. ilisha*. For the third, a hints file was generated using full-length high-quality isoform reads from long-read sequencing (PacBio RSII) from 5 tissues of *T. ilisha*. The set of potentially predicted genes was again searched for their completeness through BUSCO transcriptome analysis module^28^ against the Actinopterygii database (n: 4584). A final *de novo* predicted gene set was generated using the hints file from Iso-seq data for further genomic characterization.

The *de novo* predicted genes were annotated using Blast2GOPro suite^29^ against SwissProt and non-redundant (nr) databases, with BlastP searches (e <10^−5^). Functional domains were identified by InterProScan from Blast2GOPro suite^36^ to identify the domains and motifs of genes against different protein databases. All genes were also annotated against Kyoto Encyclopedia of Genes and Genomes (KEGG) protein database for KEGG Orthology analysis using Blastp (e <10^−5^) searches *via* KEGG Automatic Annotation Server (KAAS)^37–39^. The results obtained from GO and KEGG database annotations were compared and subsequently analysed for the GO related to homeostasis and osmoregulation and genes thereon. Maximum copy number of claudins, aquaporins, gap junction/connexins, adenylate cyclase and solute carrier gene families in final predicted gene datasets were also identified. The software tRNAscan-s.e.m. v1.23^40^ was utilised to predict tRNA genes, with eukaryote parameters.

### Orthology analysis

In order to predict conserved and unique gene families of *T. ilisha*, the orthologous groups were identified using the model species, *Danio rerio* and the migratory fishes, through Orthofinder^41^. Complete protein sequences of 11 fish species (*Clupea harengus, Cyprinus carpio, Danio rerio, Dicentrarchus labrax, Esox lucius, Gasterosteus aculeatus, Lates calcarifer, Mororns axatilis, Oncorhynchus mykiss, Oreochromis niloticus* and *Salmo salar*) downloaded from NCBI (https://www.ncbi.nlm.nih.gov/) were clustered with *T. ilisha* predicted genes, using standard default parameters (Suppl. Table S1).

### Comparative analysis of conserved regions with other species

As the chromosome level genome structure was not available in any closely related species, comparisons of *T. ilisha* draft genome were performed by mapping *T. ilisha* contigs (length>100 Kbp) to the model fish, *D. rerio* (assembly GRCz11,ftp://ftp.ncbi.nlm.nih.gov/genomes /all/GCF/000/002/035/GCF_000002035.6_GRCz11/GCF_000002035.6_GRCz11_genomic.fna.gz) using SyMap 4.2^42^ program and to *C. harengus*. At the first step, genomic sequences were aligned using promer/MUMmer^43^ and raw anchors thus obtained were grouped into gene anchors (putative), filtered with a reciprocal top-2 filter to get input to the synteny algorithm^42^. As the genome assemblies of both the *T. ilisha* (present study) and its related species, *C. harengus*, are in draft level assembly, the fragments larger than 5 Mbp were included, for meaningful results from synteny analysis.

### Characterization of complete mitogenome of *T. ilisha*

The resulting contigs from the assembled draft genome were manually searched for the presence of mitochondrial regions in BLAST^26^ searches against databases of mitochondrial sequences. The complete mitogenome of *T. ilisha* was further characterized using MitoFish and MitoAnnotator tool^44^.

To summarize, complete workflow for *T. ilisha* genome assembly and analysis has been shown in Supplementary Fig S1.

### Ethical approval

All the protocols followed were approved by Institute Animal Ethics Committee (IAEC), ICAR-NBFGR vide No. G/CPCSEA/IAEC/2015/2 dated 27 Oct., 2015. This article does not contain any studies with live animals performed by any of the authors. All the samples, that were dead, were obtained from the commercial catches. All methods were performed in accordance with the relevant guidelines and regulations.

## Results

### Genome sequence data and genome size estimation

Through the PacBio long read sequencing approach, a 125.75x coverage (104.0 Gbp) sequence data of *T. ilisha* genome was generated from 10 and 20 Kb libraries. The sub-read analysis of raw PacBio reads showed N_50_ read length of 9,181 bp, mean read length of 6,475 bp and mean read quality score of 0.87. There were a total of 9 million subreads with sizes more than 1 Kbp (Suppl. Table S3). The short read (Illumina) libraries generated reads of 88x fold (58.70 Gb) genome coverage (Suppl. Table S3), which amounted to 501 million high-quality filtered and trimmed reads (Suppl. Table S4). The genome size was estimated as 827 Mbp, by Kmer analysis, using Illumina reads and 5.3 × 10^10^ (=53754844564) Kmers were found that peaked at an index value of 65.

### Transcriptome data generation

Isoform sequencing (PacBio) of five tissues generated 29.0 Gb data, which included an average of 34,477 high quality full length read / tissue and range of 17429 to 57680 reads (Suppl. Table S5). Concatenating full-length reads from all the tissues resulted in a total of 172,388 reads, which were used to generate the of hints file for *ab initio* gene prediction.

### *De novo* assembly of genome and quality assessment

Using long reads sequences from PacBio, the assembly statistics of 5 different assembly runs on FALCON based on varying parameters showed the resultant draft assembly sizes ranged from 757.1 to 780.5 Mbp. The primary genome assembly_5 of 762.6 Mbp size with 3052 contigs, with largest contig 14.5 Mbp, N_50_ 2.65 Mbp and L_50_ 83, showed best BUSCO results (85%) for genome completeness (against the Actinopterygii gene set) and was selected for further self-correction using Quiver (Suppl. Table S5). Besides the primary assembly of 3,052 contigs, an alternate haplotypes assembly of 4,046 contigs (146.3 Mbp) was also obtained. After error correction with Quiver and Pilon, final improved primary genome assembly of 2,864 contigs showed a total size of 762.5 Mbp, which is 92.2% of the estimated genome (Table 1) on which 98.85% of the total short reads (578 million reads) were successfully mapped (Suppl. Table S7). QSTAT analysis revealed the final draft assembly of 762.5 Mbp size, with an N_50_ of 2.6 Mbp, L_50_ of 83 contigs, GC content of 43.34% and largest contig length of 17.4 Mbp (Table 1). There were only 3 contigs below 1 Kbp and only one contig below 500 bp.

**Table 1:** The quality statistics of the assembled genome derived from QUAST analysis of *Tenualosa ilisha* draft genome assembly.

Contigs (number, >= 0 bp)	2864
Contigs (number, >= 1000 bp)	2861
Contigs (number, >= 5000 bp)	2544
Contigs (number, >= 10000 bp)	2125
Contigs (number, >= 25000 bp)	1347
Contigs (number, >= 50000 bp)	996
Total length (>= 0 bp)	762512129
Lengths of contigs (>= 1000 bp)	762510094
Lengths of contigs (>= 5000 bp)	761416076
Lengths of contigs (>= 10000 bp)	758288005
Lengths of contigs (>= 25000 bp)	745674646
Lengths of contigs (>= 50000 bp)	733352674
Lengths of contigs (>= 500 bp)	2863
Largest contig (bp)	17427296
Percentage of GC	43.34
N_50_ (bp)	2624235
L_50_ (bp)	83

**Figure 1:**
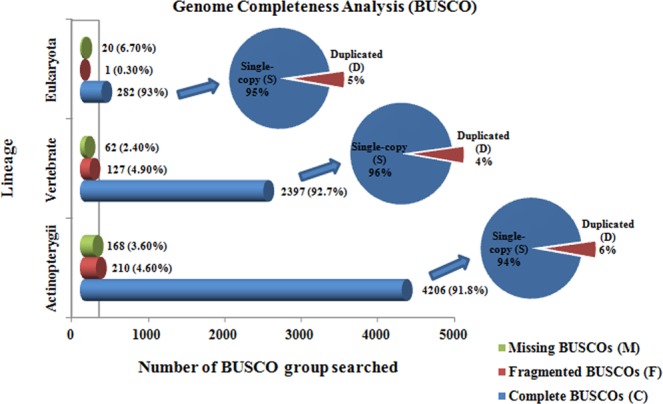
BUSCO analysis to assess the completeness of *T. ilisha* draft genome in comparison to highly conserved core genes of Actinopterygii, Eukaryota and Vertebrata.

A genome completeness analysis of the final draft assembly of *T. ilisha* against 3 BUSCO databases (Eukaryota, Actinopterygii and Vertebrata) showed a high estimate of genome completeness i.e., 96.4% (91.8% complete + 4.6% fragmented BUSCOs), with only 3.6% missing BUSCOs (Fig. 1). The detailed statistics with 3 analysed BUSCO databases are given in Suppl. Table S8.

### Repeat elements

A 12.92% of the present draft genome assembly of *T. ilisha* contain repetitive elements, which is about 90.63 Mbp and both tandem and interspersed repeats (Suppl. Table S9) were observed, among them majority are microsatellites (64 Mbp; 8.39%). A total of 1,785,618 SSRs were obtained across the genome (Suppl. Table S10). Out of total 2,867 contigs, 2,196 (76.5%) were found to contain SSR and 2,038 of these had more than one SSR. Among these identified SSRs, di-nucleotide SSRs (84.6%) were most abundant (1,977 SSR/Mbp) and densely placed (3,954 bp/Mbp) (Suppl. Fig. S2). Two contigs contain telomeric repeats (TTAGGG)_n_, covering 13.4 Kbp. These two contigs are contig00000F841; total length of 70.737 Kbp and contig00000F1661; total length of 17.437 Kbp. Transposable elements were found to belong to 14 super-families, covering 1% genome (Suppl. Table S11). Details about other classes of transposable elements and tRNA are given in Suppl. Tables S12, 13.

### Gene prediction and functional annotation

**Figure 2:**
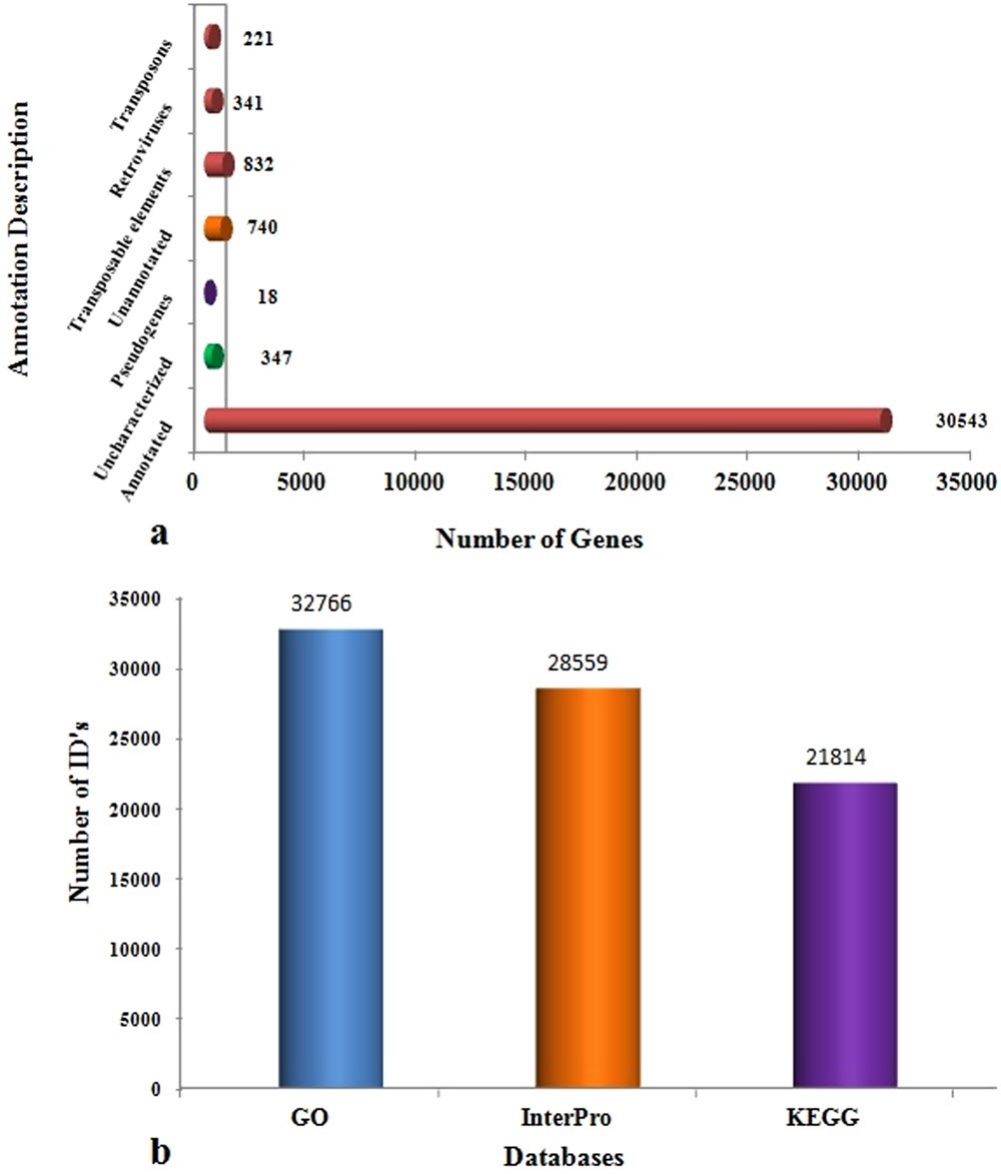
Number of Annotation of the predicted genes from *T. ilisha* draft genome (**a**) under different categories and (**b**) through different databases.

**Figure 3:**
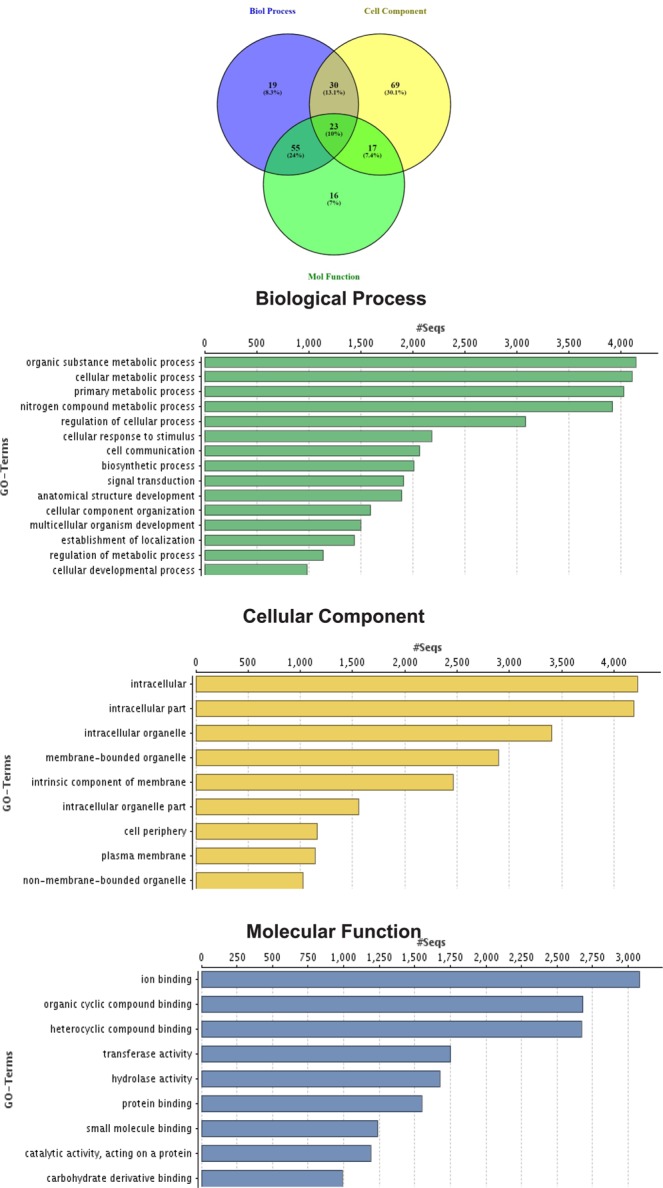
Gene Ontology (GO) functional annotations of predicted genes represented by three categories- 8831 genes were assigned with GO terms in Biological process, 9185 genes in Molecular Function and 8106 genes in Cellular component. Each category is sub-categorized in different GO terms, represented on y-axis and numbers of genes are shown in x-axis.

Evidence-based *ab initio* gene predictions with Augustus predicted 33,042 protein coding genes and BUSCO results indicated 95.8% (87.1% Complete and 8.7% Fragmented) of the core genes to be present (Suppl. Table S14).And 96.66% (31,937) predicted genes were annotated with both the databases (nr and SwissProt). The remaining 1.05% (347) genes were uncharacterized/unidentified protein families and 0.05% (18) pseudo genes, while 2.2% (740) genes remained unannotated (Fig. 2: Suppl. Table S15). Analysis of Gene Ontology (GO) terms revealed that GO terms were assigned to 32,766 genes and 4.9% of GO terms were found to be unique to biological processes, 10.2% in cellular functions and 10.6% in molecular processes, while 43.9% were shared by all the three categories (Fig. 3). InterPro analysis revealed 28,559 genes with significant hits, while the high number of genes was associated with protein families from the PFAM database (26,582), as compared to other four databases.

**Figure 4:**
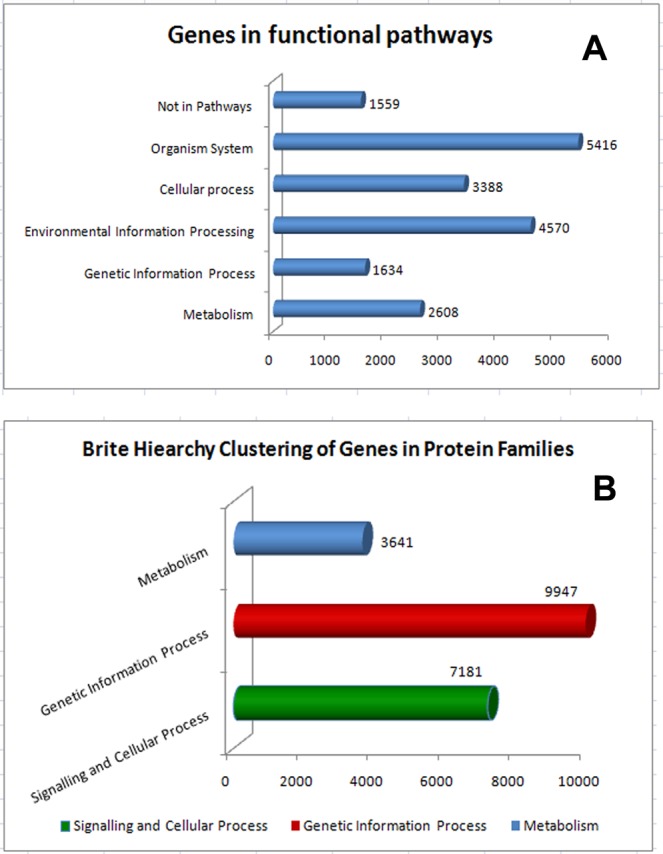
(**A**) Genes categorized into different pathways in KEGG’s analysis. Majority of genes categorized into Organismal System (GIP) pathways of KEGG’s orthology. (**B**) Brite hierarchy clustering of KEGG annotated genes into different protein families.

The KEGG analysis classified sequences majority into three protein families, metabolism (3,641 genes), genetic information processing (9,947) and signaling and cellular process (7,181) (Fig. 4). A total of 22,549 genes in KEGG pathways were assigned with 8,6147 unique KEGG Orthology (KO) numbers. These genes were functionally categorized into 5 major KO categories: Metabolism (2608 genes; 13%), Genetic information processing (1634; 8%), Environmental information processing (4570, 24%), Cellular processes (3,388; 18%) and Organismal Systems (5416, 28%). However 1659 (9%) genes with KO terms could not be assigned to any major categories. The GO and KO results were further mined for the genes related to osmoregulatory processes.

### Genes associated with homeostasis

**Figure 5:**
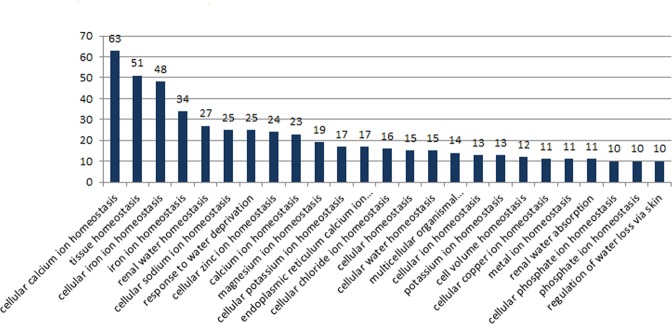
Genes associated with homeostasis functions categorized through Gene Ontology (GO) analysis in from total genes predicted from *Tenualosa ilisha* genome assembly.

**Figure 6:**
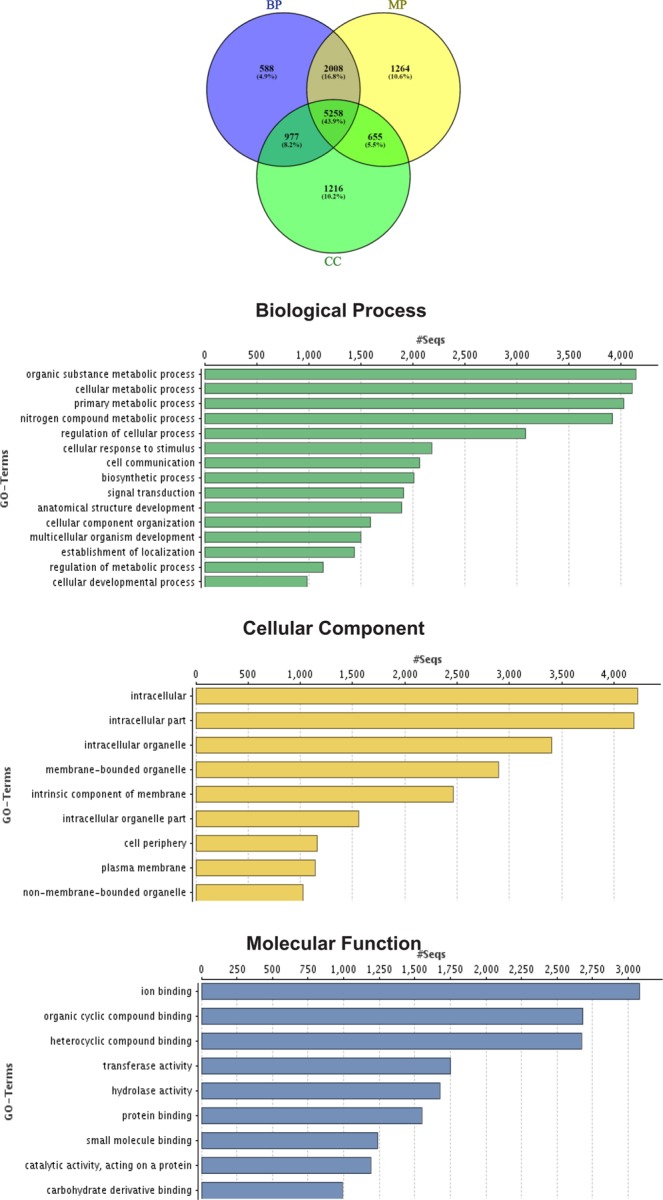
(**A**) Vann diagram showing GO Functional annotations of homeostasis related genes that were unique for and shared by three categories. (**B**) Gene ontology terms for (a) biological process (b) cellular components (c) molecular function for top 50 GO Terms from 512 genes with homeostasis functions in *Tenualosa ilisha*.

On the basis of functional annotations, 61 GO terms were found to be associated with homeostasis and included 512 genes (Figs 5, 6; Suppl. Tables S16, 17), which include ion and water homeostasis. Among these, highest number of genes (63) were under cellular calcium ion homeostasis (GO: 0006874), in which there were 9 copies of Kinase C-beta type gene, while 6 copies each were found for glutamate receptor kainate 2 and sodium potassium calcium exchanger 3 (Suppl. Table S18). Next highest number (51) of genes was found in GO:0001894: tissue homeostasis, includes, besides many other genes, highest number of 6 copies for CYR61 and 5 for COL2A1 genes (Suppl. Table S19). A total of 48 genes was categorised under cellular iron ion homeostasis (GO:0006879), majority of which are ATP-binding cassette Sub-family B and G members and Transmembrane protein serine 6 and 9, with multiple copies (Suppl. Table S20). Under water homeostasis, 97 genes were found, under which important GO: renal water homeostasis (GO:0003091) contains 27 genes, followed by response to water deprivation (GO:0009414, 25 genes), cellular water homeostasis (GO:0009992, 15 genes) and renal water absorption (GO:0070295, 22 genes). Renal water homeostasis (GO:0003091) also includes eight genes of adenylate cyclase, with multiple copies, highest of which is of ADC48 (9 copies) (Suppl. Table S21). Genes identified under water homeostasis are given in Suppl. Table S21.

**Figure 7:**
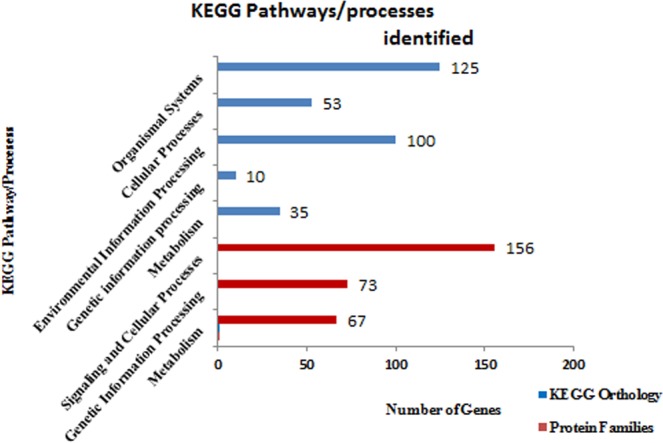
KEGG pathways/processes identified for homeostasis functions in *Tenualosa ilisha*.

Among these 512 genes with GO terms related to homeostasis, analysis of KEGG pathways resulted in majority (125) genes under organismal systems, followed by environmental information processing (100) (Fig. 7; Suppl. Table S22). Lipid and carbohydrate metabolism contained maximum number of genes (10 each), while signal transduction pathway (89 genes) and signalling molecules and interaction (30 genes) came out as the most crucial pathways in *T. ilisha* homeostasis. Signal transduction pathways includes Ras signalling pathway [PATH:ko04014] with 13 genes (Suppl. Fig. S3), followed by Rap1 signalling pathway [PATH:ko04015] with 10 genes (Suppl. Fig. S4). Other pathways with 7 genes each were Calcium signalling pathway [PATH:ko04020] (Suppl. Fig. S5) and FoxO signalling pathway [PATH:ko04068]. It was interesting to find four genes, i.e., PIK3CA_B_D (phosphatidylinositol-4,5-bisphosphate 3-kinase catalytic subunit alpha/beta/delta) gene with 4 copies, PRKCA (classical protein kinase C alpha type, 2 copies), PKA (protein kinase A, 4 copies) and CAMK2 (calcium/calmodulin-dependent protein kinase (CaM kinase) II, 4 copies) and adenylate cyclases (ADCY1,2,3,6,8,9) participate in almost all the signalling pathways.

Digestive (54 genes) and endocrine (48 genes) system were prominent among the Organismal Systems pathways. In digestive system, genes of osmoregulatory nature were placed among bile secretory pathway (16 genes), gastric acid secretion (23 genes) and bile secretion (17 genes) (Suppl. Table S23), among which membrane bound proteins like solute carriers, ATP4B (H+/K+-exchanging ATPase subunit beta), Aquaporins and ATP-binding cassettes (MRPs) were found. Under the endocrine system, the pathway of Parathyroid hormone synthesis, secretion and action has maximum (23) number of osmoregulatory genes (Suppl. Table S25). While in excretory system (Suppl. Table S25), pathways like Aldosterone-regulated sodium re-absorption, endocrine and other factor regulated calcium absorption and vasopressin-regulated water re-absorption play major roles, with 9 genes in each pathway, where ATP1B/CD298 (sodium/potassium-transporting ATPase subunit beta, 5 copies) being prominent in 4 pathways. Pathways for communication between the cells, which include Focal adhesion [PATH:ko04510], Adherens junction [PATH:ko04520], Tight junction [PATH:ko04530] and Gap junction [PATH:ko04540], contain 34 homeostasis genes.

In addition, among the homeostasis processes, largest number of genes belonged to Adenylate cyclase (ACD, 8 genes with total 22 copies), Aquaporins (AQP, 9 genes, 21 copies), solute carrier gene (SLC, 16 families, 34 genes, 61 copies) and voltage gated potassium channel gene (45 genes, 135 copies) families (Suppl. Table S26). The present study also identified a considerable number of claudins (CLD) encoding genes (23 genes, 68 copies) and gap junction/connexin (CX) coding genes (21 genes, 55 copies) in the *T. ilisha* genome assembly.

### Conserved and unique gene/protein families

**Figure 8:**
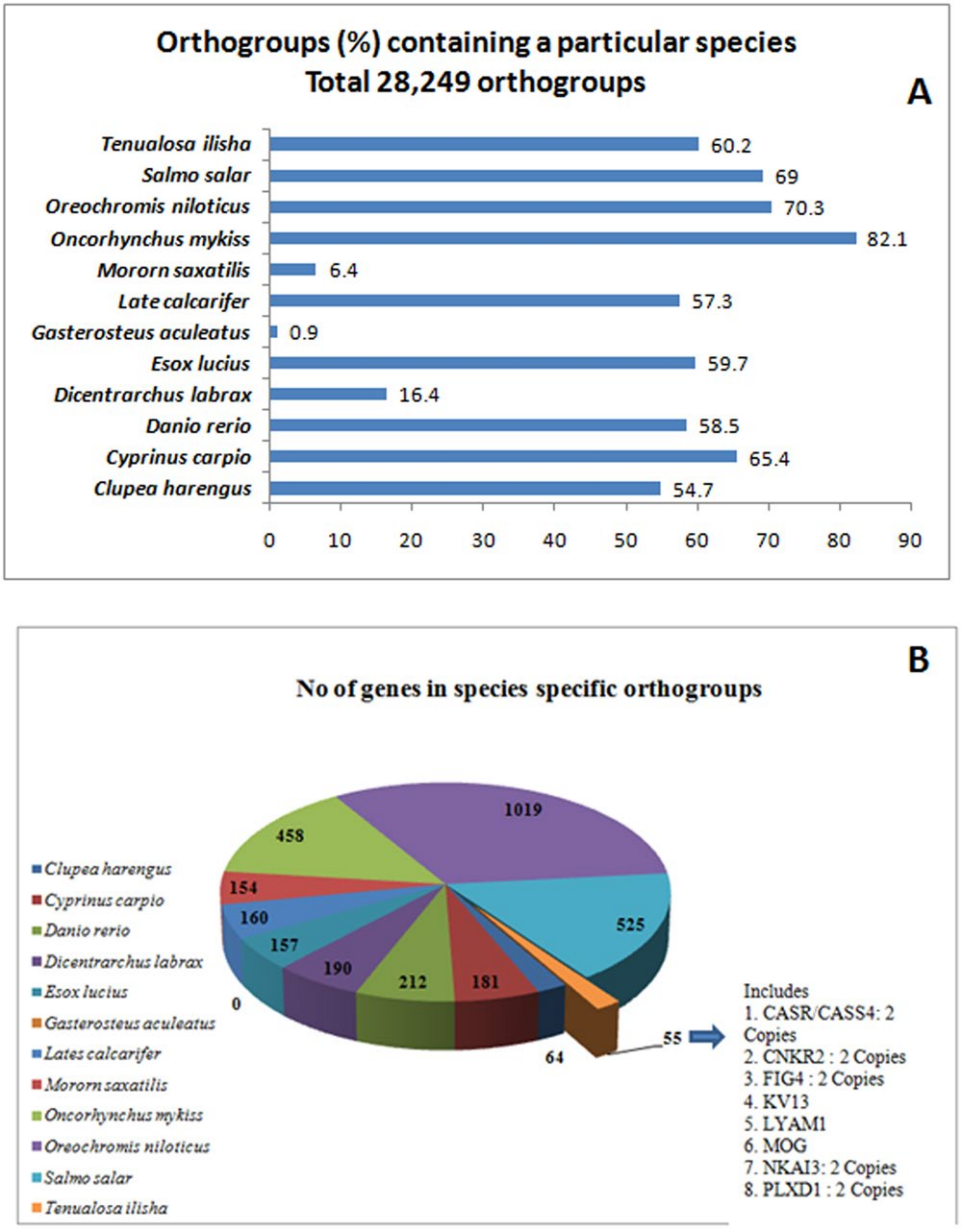
(**A**) Common orthogroup from the comparison of total proteins from 12 fish species, including *Tenualosa ilisha*. (**B**) Species specific orthogroup in *Tenualosa ilisha*, which included novel variants and new genes.

Among the 12 analysed fish proteomes, including that of *T. ilisha*, a total of 28,249 common and 518 species specific orthogroups were identified among all selected species (Suppl. Table S27). Proteins from *T. ilisha* were included in 17,015 common orthogroups (Fig. 8a) and *T. ilisha* specific 18 orthogroups contain 55 proteins (Suppl. Table S28).

Among these 55 proteins (Table S29), interestingly 2 genes, NKAI3 (Sodium potassium-transporting ATPase subunit beta-1-interacting 3) and L4AM1 (L-selection) are reported here, for the first time in fish. The remaining are novel variants of genes, already known in literature, which included variants for seven genes (Fig. 8b; Supp. Table S30). Among them, CASR/CASS4, CNKR2 and FIG4 have 2 copies each, while KV13, LYAM1, MOG and PLXD1 one each.

Orthology analysis of four (ACD, AQP, CLD, CX) osmoregulatory related gene families with 43 other fish species (Suppl Table S2**)** revealed that most of the fish species have highly similar orthogroups, based on these genes. Comparison of claudin genes of 43 fish species revealed that majority of the species have all their Claudin genes designated in orthogroups. However, one species-specific orthogroup of *T. ilisha* emerged, which contained 2 CLDZ genes. For other three gene families, no species-specific *T. ilisha* orthologous group was observed, although, sufficient number of fish species was used for analysis.

### Comparative genomics through synteny analysis

The synteny analysis of *T. ilisha* draft genome and chromosomal level assembly of the model fish species, *Danio rerio* resulted in mapping of 660 sequences from *T. ilisha* (>100 Kbp size) over *Danio rerio* (25 chromosomes), with 174 synteny blocks (Suppl. Fig. S6). Further, comparisons of *T. ilisha* assembly with its closest species, *C. harengus*, showed the mapping of a total of 19 sequences of *C. harengus* on 23 of *T. ilisha* (36% mapped) (Suppl. Table S30, Fig. S7). Maximum hits (1,051) were found between *T. ilisha* contig_2778 and *C. harengus* contig_2741, with Pearson R value of 0.99919 (Suppl. Fig. S8).

### Mitochondrial genome of *T. ilisha*

The complete mitogenome of *T. ilisha* was found to be composed of one contig, of length of 16,745 bases, contains 13 protein-coding genes. Non-protein coding regions include 2 ribosomal RNA, 22 tRNAs and a D-loop region (Suppl. Table S32). The genes arrangement was found to be typical of fish/vertebrate mitogenomes. All the genes were encoded on the heavy strand, except for ND6 and eight tRNA genes.

## Discussion

The present study reports the first high-quality draft genome assembly of Hilsa shad, *T. ilisha*, from the family Clupeidae (Order Clupeiformes). The estimated genome size of 827 Mbp is in good agreement with that of another member of Clupeidae family, *Clupea harengus* (808 Mbp)^45^ and other herrings (http://www.genomesize.com).

We have successfully generated *T. ilisha* draft genome assembly of 2,864 highly contiguous contigs (762.5 Mbp) with a high N_50_ than reported in earlier studies^46,47^. Moreover, the genome assembly from the present study shows a high completeness and its GC% is similar to that reported for other teleosts^45,48,49^. In addition, it shows high synteny and co-linearity with the model species, *D. rerio* as well as with *C. harengus*. However, it was surprising to find the repeat content in *T. ilisha* to be significantly lower than that in *C. harengus*, which may be due to absence of tandem repeats rich telomeric regions^50^ in the present genome assembly. However, the high percentage (95.8%) of core genes suggests its high level completeness of euchromatin components. In addition, the long reads hint file efficiently predicted a high proportion of full-length genes, as accurate start and stop codons were made available for gene predictions.

Interestingly, the *T. ilisha* specific orthogroup, with 55 gene sequences, contains two genes, NKAIN3 and L4AM1, which are reported here for the first time in fish. NKAIN3 has been reported as a family of mammalian proteins with an orthologue in *Drosophila melanogaster*^51^. Its functions are yet to be defined, but its important role for neuronal function by interacting with the beta subunit of Na,K-ATPase is speculated^51^. In human*, NKAIN3* gene is reported to express in foetal temporal lobe of the brain^51^ and its variant was found to be associated with cognitive traits. L4AM1 (L-selectin, also known as CD62L) belongs to a family of adhesion/homing receptors and is responsible for Ca^2+^ ion dependent cell to cell adhesion^52^. Along with the gap junctions, it links plasma membranes of adjacent cells^53^.

Among *T. ilisha* specific orthogroup members, the genes, for which novel variants are identified, are involved in signalling processes, i.e.  FIG4 ^54^ and Plexin D1^55^ play key roles in vesicle trafficking and latter in regulating the migration of wide spectrum of cell types, respectively. CASR is a receptor for sensing the signal of calcium ion entracellular levels, controls calcium homeostasis, by regulating the release of parathyroid hormone^56^. CASS4 has a role in signalling related to cell and focal adhesion integrity^56^ and KV13 mediates voltage dependent ion membrane permeability (https://www.ncbi.nlm.nih.gov/gene/3738). CNKR2 mediates the mitogen-activated protein kinase pathways downstream from Ras (https://www.genecards.org/cgi-bin/carddisp.pl?gene=CNKSR2), while MOG (Myelin-oligodendrocyte glyco protein) mediates homophilic cell-cell adhesion^57^.

Fish have evolved the mechanisms to face the challenges, due to the aquatic environment, for maintaining their internal ion homeostasis, against the external ionic gradients. Although the kidneys form a major organ for osmoregulation, the integument, gills and intestine also play important roles, to maintain ion and water homeostasis^9^. In general, euryhaline fishes adopt the strategy of dynamic control through hypo- and hyper-osmoregulation^9^.

In hilsa shad, on the basis of their functions related to homeostasis, we found highest number of genes, for cellular calcium ion homeostasis, as compared to other GOs. Fish fulfills all its requirement of Calcium (Ca^+2^) from water and many fish species are known to adjust to varying calcium concentrations in water. Among the genes under this category, the highest number of copies were found for Kinase C-beta type (PRKCB) gene (https://www.ncbi.nlm.nih.gov/gene/5579), followed by sodium potassium calcium exchanger 3 (NCKX3, SLC24A3). PRKCB is a member of serine- and threonine-specific protein kinases family and is involved in expression of Claudin proteins, which are responsible for tight junction membrane permeability^58^. NCKX3/ *SLC24A3* functions in the transport of intracellular calcium across the cell membrane in exchange for extracellular sodium ions and is expressed in many tissues, including, intestine^59^, as well as in kidney^60^ for osmoregulation of body fluids. Pinto *et al*.^61^ studied gill transcript expression patterns in green spotted puffer fish for different [Ca^2+^] and suggested occurrence of cytoskeletal proteins active re-modeling during the initial acclimation process.

Another class of genes, ATP-binding cassette or ABC, identified under cellular iron ion homeostasis in *T. ilisha* were found in multiple copies. These are trans-membrane transporter proteins and with the help of ATP, are involved in transportation of several types of molecules across cellular membranes^62^. In human, several of ABC proteins have been reported to involved in lipid homeostasis^63^. In trout, multiple copies of trans-membrane protein serine genes were reported and ATP-binding cassette sub-family F member 1 (ABCF1) was found to be up-regulated in freshwater ionocytes^64^. In tissue homeostasis, presence of multiple homologues of CYR61 (CCN1) was reported and was predicted to be involved in cell adhesion and negative regulation of cell death^65^ in zebra fish.

The kidney is an important organ in euryhaline fish, for ion homeostasis and osmoregulation of body fluids during the process of adaptation to various salinities^66^. In Hilsa, Under water homeostasis category, highest number of genes were found in renal water homeostasis, which may be the indication of importance of this process in Hilsa. Adenylate cyclases are known osmosensors and their role in osmoregulation is well known^67^ (discussed in detail in later section). Prolactin, a pituitary hormone, is known to be required for fresh water-adaption in fish^68^. Hormones of Vasopressin-V2-type receptor-aquaporin axis control the extra- and intra-cellular fluid osmoregulation^69^.

The present study observed AQP, SLC and voltage gated potassium channel gene families in multiple copies, which are related to homeostasis, including osmoregulation. The indirect role of adenylate cyclase and aquaporins, as osmosensors, has been reported in euryhaline fishes for regulating osmoregulatory mechanisms against salinity stress^67^ and were suggested to have arisen from the early duplication events of the ancestral genes^70^. In Atlantic salmon, six isoforms of Aquaporins ie., AQP-1a,b; 3a; 8a,b and 10a were differentially expressed in different salinity levels in gill and intestine, however, AQP-8b was suggested to be a key water channel responsible for water uptake in the intestinal tract of seawater salmonids^71^.

Solute Carrier (SLC) transporters are secondary transporters, which control trans-membrane movement of important substrates and 338 putative genes in 50 families are reported in fishes, which are important for cellular influx and efflux^72^. Out of eight transporter families reported to be involved in the processes to enable fish to tolerate different salinity levels^72^, Hilsa possesses six families. Four members of Na+/(Ca2+-K+) exchanger family, SLC24 (homologue NCKX in human), i.e. a1, a3 and a5^72^ and a2^73^ have been reported in fish. It is interesting to find the presence of 4 members, a2, a3, a4 and a6 in Hilsa, of which a6 is being reported for the first time in a fish species, whose homologues is found in human^74^. SLC24a6 has been reported to play a role in different tissues and cell types ^for^ K^+^-dependent Na^+^/Ca^2+^ exchange in maintaining cellular Ca^2+^ homeostasis^75^. Further, SLC5a8, 34a, 6a15, 2a5, 43a3 and 39a4 were reported to be responsive to osmoregulation as well as salinity stress in the liver of spotted sea bass^76^ and SLC13a1 and SLC26a1 to freshwater osmoregulation^77^

Proteins involved in K^+^ voltage-gated channels are also found in multiple copies in the present study. These are reported to be responsive to changes in voltage of cell membrane and are involved in several functions, including transportation of K^+^ across basolateral membranes^78^ and volume regulation^79^. However, not much is known about their detail functions in teleosts. These channel transcripts have been detected in the intestinal epithelium^80^ and in gills^81.82^ from various fish species. In Atlantic Salmon gills, regulation of expression of BK channel during acclimation to different salinity levels suggested their possible role in osmoregulation^81–83^. In Hilsa, involvement of a particular gene(s), from the multiple genes reported here, can be pinpointed after the experimentation with different salinity levels.

Claudins and gap junction/connexin proteins encoding genes are involved in tight and adherens junctions of epithelial cells and control the exposed epithelia permeability at the time of salinity changes^15^. Their high copy number may relate to the modulation of activities required to adjust to the dramatic changes, controlling tight junctions, during fish adaptation to varying osmotic and ionic gradients^15^. The presence of multiple claudins in *T. ilisha* (68) exceeds the number of previously reported in European seabass (61), stickleback (57), tilapia (56), fugu (56), zebrafish (54), medaka (48) and Atlantic cod (47)^11,15,84^. Role of isoforms of Claudin 6, 8, 10, 15 and 27 have been reported for acclimation to freshwater and seawater adaptation in fish^85–88^. Orthology analysis of specific osmo-regulatory genes revealed a variant of Claudin in *T. ilisha*, CLDZ gene with two copies, which has been reported to be responsive to nociceptive events in fish^89^.

Likewise, a larger number of connexin protein-encoding genes are represented in the present genome (53) compared to 39 to 40 reported in other teleost fishes, i.e., zebrafish, herring, catfish, fugu, tilapia, and medaka^90,91^. Connexins are integral membrane proteins that oligomerize to form gap junctions (proteinaceous channels) that permit the transfer of small molecules (<1 kDa) between neighboring cells. It is interesting to find presence of Cx33, which is single “mouse-specific” connexin^92^ and is a testis-specific gap junction protein^93^. It is absent both from the human genome and zebrafish genome. Cell adhesion and tight junction proteins are quickly up-regulated in response to hypo-osmotic shock and transcripts for proteins connexin-32.2, claudin-3, and claudin-4 were reported to be up-regulated in Killifish^94^.

In *T. ilisha*, the presence of multiple copies of genes may suggest expansions of these genes for adaptation to extreme environmental conditions, independent from the proposed whole genome duplication in teleost fish^14^, and may provide the genomic landscape for the anadromous lifestyle in *T. ilisha*. Moreover, adaptation by a euryhaline fish to changing environmental salinity is an energy expensive process and for iono- and osmo-regulation, metabolic pathways plays a significant role in the energy supply^95^. In present study, homeostasis genes were also found in Carbohydrate and lipid metabolism pathways, which play a significant role in energy supply. As a migratory fish requires lower energy in acclimation to low salinity water than that in high salinity^96^, these metabolic pathways may play a significant role in hilsa migration from fresh to marine environment for growth.

To sense the changing environment, *T. ilisha* seems to transduce stimulus to signalling pathways, which in turn activates the specific changes required for osmoregulation, as signal transduction pathway and signalling molecules and interaction seems to be the most crucial pathways in *T. ilisha*. Differential response of cell stress pathway genes, triggered by osmotic change, results in up-regulation of cell cycle and signal transduction has been reported in euryhaline fish^97^. Among the organismal systems pathways, involvement of membrane bound proteins with homeostasis function seems most prominent in digestive and excretory system of *T. ilsha*, while in endocrine system, pathways supporting for ion- and water reabsorption seems significant. This is quite reasonable to consider that under various osmotic conditions, the upkeep of intracellular homeostasis necessitates significant activities by these trans-membrane systems.

## Conclusions

The assembly and analysis of the genome of an organism provides a most important link to understanding the biology, ecology and adaptations of that species. Although the draft genome of *T. ilisha*, in the present study, is fragmented, however it is highly contiguous with a high N_50_ than that reported in earlier studies^46,47^. This high-quality draft genome with nearly complete euchromatin and predicted genes, especially those with homeostasis and osmoregulatory function, form an important genomic resource in the species. This resource can accelerate scientific investigations of important traits in Hilsa, particularly for its adaptive mechanisms for facing varying salinity levels during the migrations.

## Supplementary information

Supplementary Figures

Supplementary tables

## Data Availability

The raw PacBio reads are available through the Short Read Archive (SRA) database (SRP126802) of NCBI under Bioproject (PRJNA422030) and Bio-Sample (SAMN08162886). This Whole Genome Shotgun project has been deposited at DDBJ/ENA/GenBank under the accession PYXC00000000. The version described in this paper is version PYXC01000000, includes primary and alternate contigs. The raw sequence reads from Illumina sequencing were submitted to NCBI, SRA database (SRR 6384292, SRR6384293 and SRR6384294) under Bioproject (PRJNA422030) and Bio-Sample (SAMN08195513). The raw iso-seq reads of PacBio RSII were submitted to SRA database under Bioproject (PRJNA417747). SRA and BioSample Accessions were provided in Table S5.
