# Supplementary material for: Draft genome assembly of Tenualosa ilisha, Hilsa shad, provides resource for osmoregulation studies
Source: Sci Rep. 2019 Nov 11;9:16511. doi: 10.1038/s41598-019-52603-w (PMC6848103; doi:10.1038/s41598-019-52603-w)

**Supplemental Information (Figures)**

**Draft genome assembly of *Tenualosa ilisha,* Hilsa shad, provides resource for osmoregulation studies**

Vindhya Mohindra^1*^, Tanushree Dangi^*^, Ratnesh K. Tripathi^*#^ , Rajesh Kumar^*^, Rajeev K. Singh^*^, J. K. Jena^!^, T. Mohapatra^!^

^*^ICAR-National Bureau of Fish Genetic Resources, Canal Ring Road, P.O. Dilkusha, Lucknow - 226 002 INDIA, ^!^Indian Council of Agricultural Research (ICAR), Krishi Anusandhan Bhawan - II, New Delhi - 110 012 INDIA. #Present address : Imperial Life Sciences (P) Limited, Gurgaon, Haryana 122001 INDIA

**Corresponding author** : Vindhya Mohindra

ICAR-National Bureau of Fish Genetic Resources, Canal Ring Road, P.O. Dilkusha, Lucknow - 226 002 INDIA

email: vindhyamohindra@gmail.com; vmohindra@nbfgr.res.in

**Table of contents**

| **Supplementary figures:** | **Page 2 to 7** |
| --- | --- |

**Long Read**

**Genomic Data**

**(PacBio RS II )**

**Short Read**

**Genomic Data**

**(Illumina)HiSeqq)**

**Long Read**

**Transcriptome Data**

**(PacBio RS II )**

**De Novo Assembly**

**(FALCON)**

**Primary Assembly (Contigs)**

**Error Correction**

**(Quiver)**

**Error corrected**

**Primary Assembly (contigs)**

**Error correction**

**(PILON)**

**High Quality**

**Draft Assembly**

**Basic Statistics of Assembly**

**(QUAST)**

**Genome Completeness**

**Assessment (BUSCO)**

**Genome Size Estimation**

**(K-mer Analysis)**

**Repeat Identification**

**and Characterization**

**Mitochondrial**

**Genome Analysis**

**and Phylogeny**

**Repeat masked**

**Draft Assembly**

**Gene Prediction**

**High Quality Full length Transcript**

**Iso-Seq Analysis to generate**

**High Quality Full length**

**Transcripts**

**Hints File**

**Generation**

**Annotationnn**

**Orthology Analysis**

**Synteny Analysis**

**(>100K read length)**

**Functional**

**(Gene Ontology)**

**Pathway Analysis**

**(KEGG)**

**Osmoregulatory Genes Identification, Characterization**

**Repeat masking**

**Raw Data Processing**

**(QC and Filtering)**

**Fig. S1:** Genome assembly and analysis workflow for *Tenualosa ilisha***.**

.


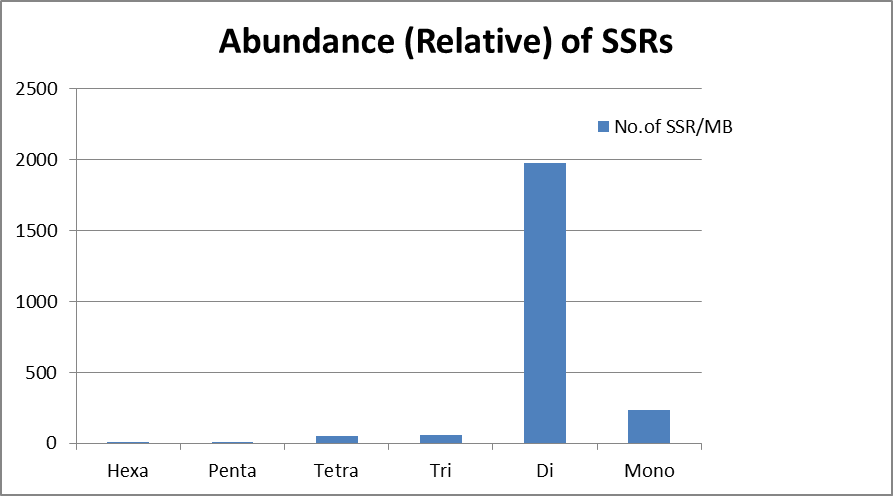


**Fig. S2.** Relative Abundance of SSRs in *Tenualosa ilisha* genome represents the abundance of Dinucleotide (1977.1 SSRs/MB).

**
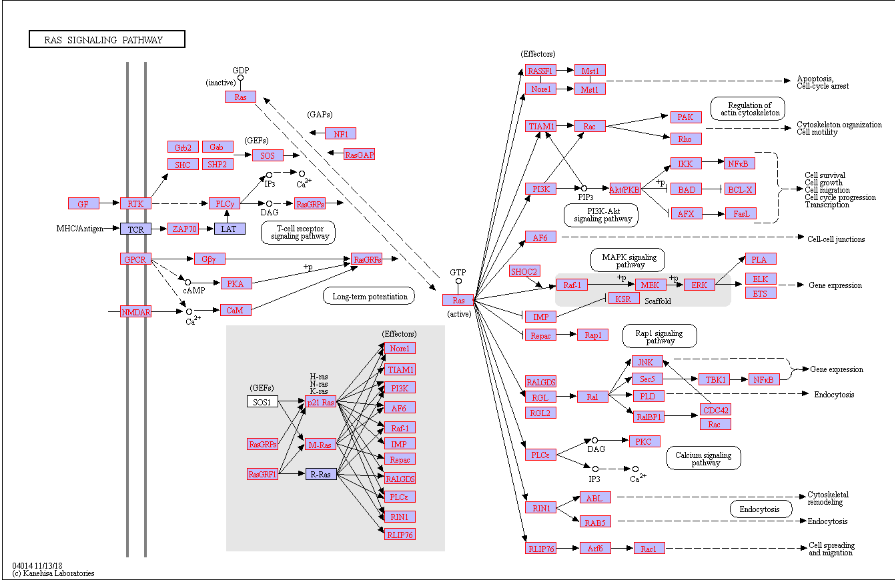
**

**Fig. S3.** Genes in Ras Signalling pathway found from osmoregulatory genes identified in *Tenualosa ilisha* (shown in red) (Reprinted with permission from Kyoto Encyclopedia of Genes and Genomes). ^37,38,39^


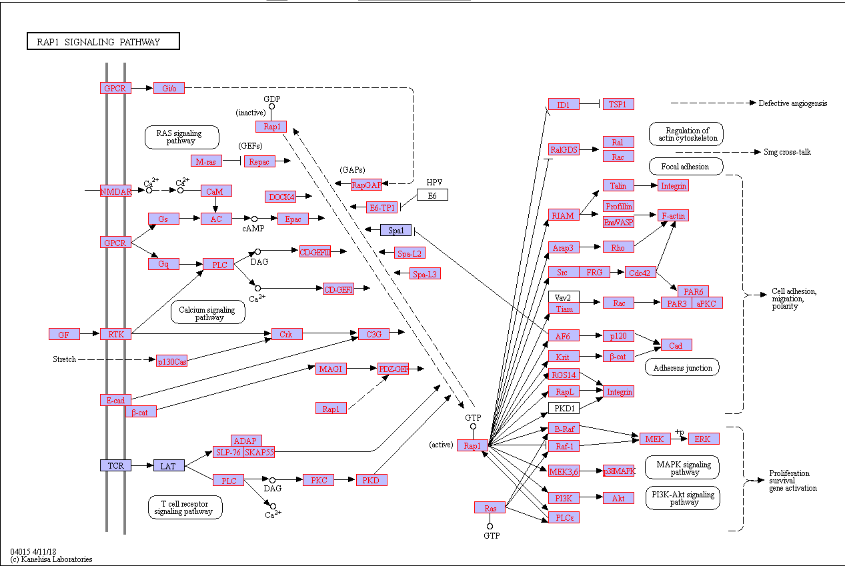


**Fig. S4.** Genes in Rap1 Signalling pathway found from from osmoregulatory genes identified in *Tenualosa ilisha* (shown in red) (Reprinted with permission from Kyoto Encyclopedia of Genes and Genomes). ^37,38,39^


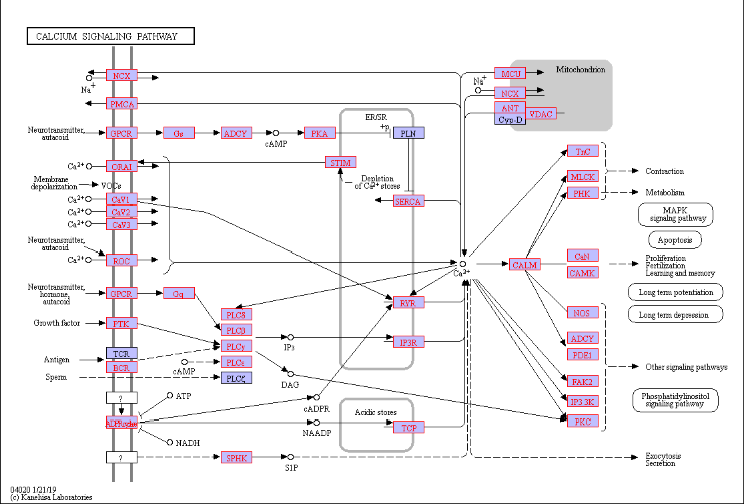


**Fig. S5.** Genes in Calcium Signalling pathway found from osmoregulatory genes identified in *Tenualosa ilisha* (shown in red) (Reprinted with permission from Kyoto Encyclopedia of Genes and Genomes). ^37,38,39^

**
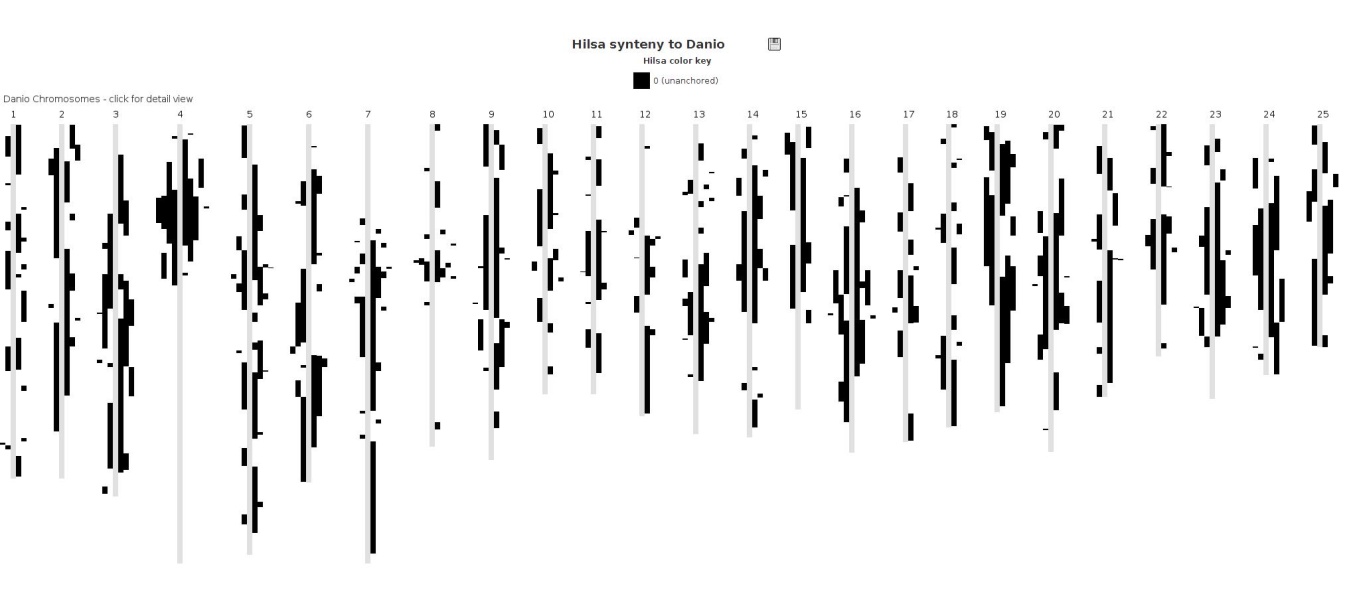
**

**Fig. S6.** The block view Diagram of Synteny analysis showing the mapping of *Tenualosa ilisha* genome (n= 174 blocks) on 25 chromosomes of *Danio rerio* (n= 343 blocks).

**Fig. S7.** The block view Diagram of Synteny analysis showing the mapping of *Clupea harengus* genome on *Tenualosa ilisha* genome .


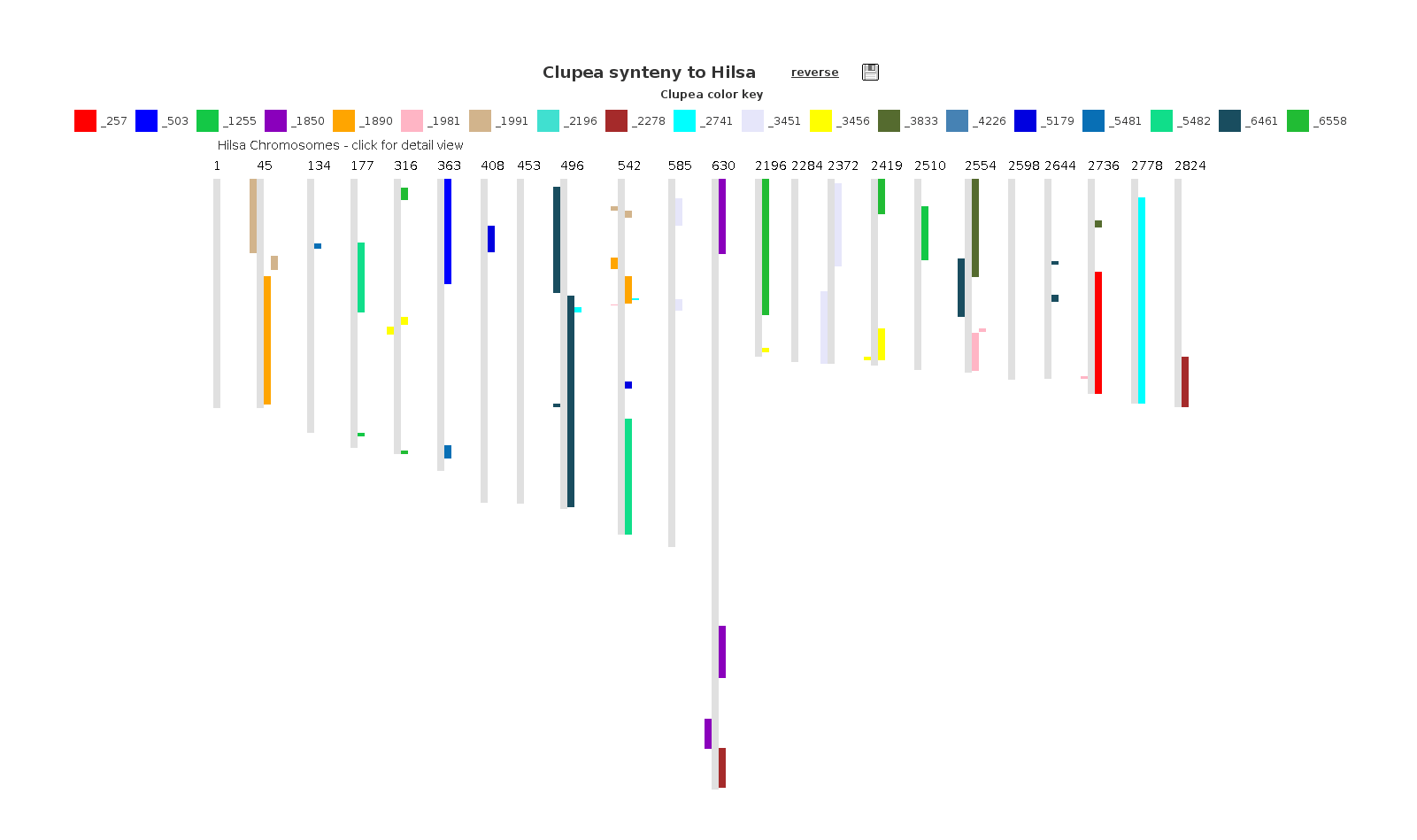


**Fig. S8.** Comparative synteny analysis of largest assembled contigs (Contig 630; 17.43 MB) of *T. ilisha* with chromosomes 7 (74.28 MB) of *Clupea harengus.*


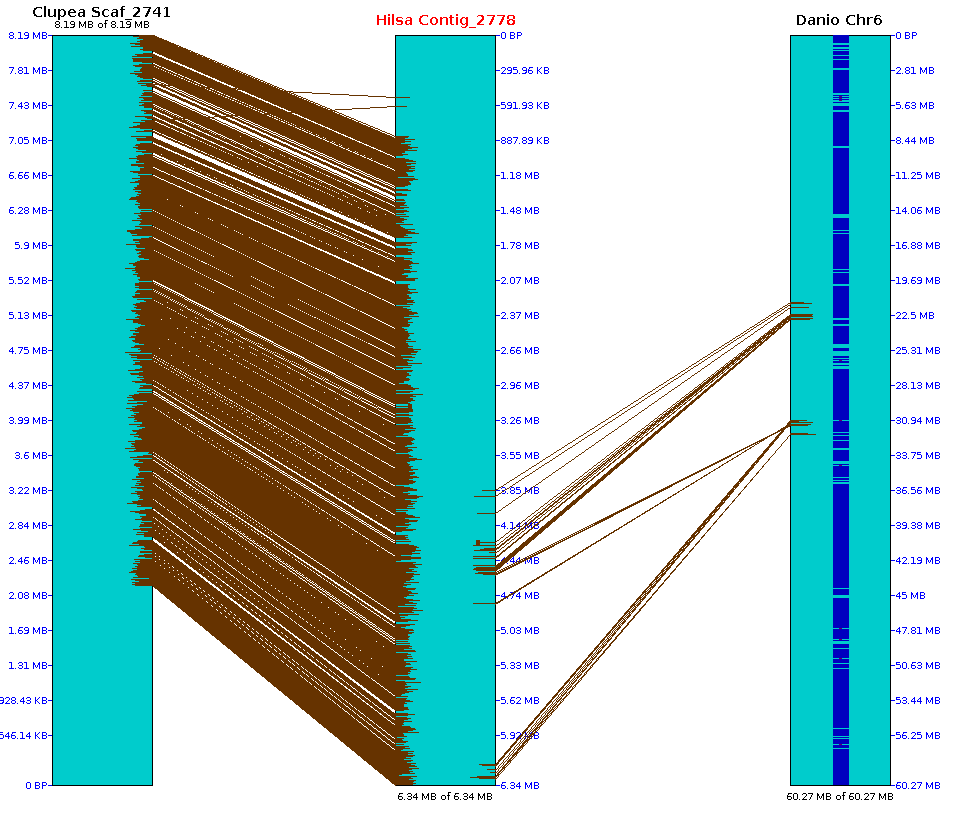

Supplement: Supplementary file 1 — Supplementary Figures [file 41598_2019_52603_MOESM1_ESM.doc]
