# Supplementary material for: Draft genome assembly of Tenualosa ilisha, Hilsa shad, provides resource for osmoregulation studies
Source: Sci Rep. 2019 Nov 11;9:16511. doi: 10.1038/s41598-019-52603-w (PMC6848103; doi:10.1038/s41598-019-52603-w)
Supplement: Supplementary file 2 — Supplementary tables [file 41598_2019_52603_MOESM2_ESM.doc]

**Supplemental Information (Tables)**

**Draft genome assembly of *Tenualosa ilisha,* Hilsa shad, provides resource for osmoregulation studies**

Vindhya Mohindra^1*^, Tanushree Dangi, Ratnesh K. Tripathi^#^ , Rajesh Kumar, Rajeev K. Singh, J. K. Jena^!^, T. Mohapatra^!^

^*^ICAR-National Bureau of Fish Genetic Resources, Canal Ring Road, P.O. Dilkusha, Lucknow - 226 002 INDIA, ^!^Indian Council of Agricultural Research (ICAR), Krishi Anusandhan Bhawan - II, New Delhi - 110 012 INDIA. #Present address : Imperial Life Sciences (P) Limited, Gurgaon, Haryana 122001 INDIA

**Corresponding author** : Vindhya Mohindra

ICAR-National Bureau of Fish Genetic Resources, Canal Ring Road, P.O. Dilkusha, Lucknow - 226 002 INDIA

email: vindhyamohindra@gmail.com; vmohindra@nbfgr.res.in

**Table of contents**

| **Supplementary tables:** | **Page 2 to 37** |
| --- | --- |

**Table S1** Complete protein sequences of fish species (n=12) used in orthology analysis, from NCBI.

| **S. No** | **Species** | **No. of Protein**  **Sequences**  **Used for Orthology**  **Analysis** | **Website Link**  **/Accession no** |
| --- | --- | --- | --- |
|  | *Clupea harengus* | 29855 | https://www.ncbi.nlm.nih.gov/protein |
|  | *Cyprinus carpio* | 64455 |
|  | *Danio rerio* | 54387 |
|  | *Dicentrarchus labrax* | 15082 |
|  | *Esox Lucius* | 65858 |
|  | *Gasterosteus aculeatus* | 4578 |
|  | *Late calcarifer* | 45752 |
|  | *Mororn saxatilis* | 14555 |
|  | *Oncorhynchus mykiss* | 144253 |
|  | *Oreochromis niloticus* | 97968 |
|  | *Salmo salar* | 116800 |
|  | *Tenualosa ilisha* | 33696 |

**Table S2. List of the species from which Claudins, Aquaporins and Connexins were used for orthology analysis alongwith that of *Tenualosa ilisha.***

| **Claudin** | **Aquaporin** | **Connexin** |
| --- | --- | --- |
| *Acanthochromis polyacanthus* | *Acanthochromis polyacanthus* | *Acanthochromis polyacanthus* |
| *Astyanax mexicanus* | *Astyanax mexicanus* | *Astyanax mexicanus* |
| *Austrofundulus limnaeus* | *Austrofundulus limnaeus* | *Austrofundulus limnaeus* |
| *Boleophthalmus pectinirostris* | *Boleophthalmus pectinirostris* | *Boleophthalmus pectinirostris* |
| *Callorhinchus milii* | *Callorhinchus milii* | *Callorhinchus milii* |
| *Clupea harengus* | *Clupea harengus* | *Clupea harengus* |
| *Cyprinodon variegates* | *Cyprinodon variegates* | *Cyprinodon variegates* |
| *Cyprinus carpio* | *Cyprinus carpio* | *Cyprinus carpio* |
| *Danio rerio* | *Danio rerio* | *Danio rerio* |
| *Esox lucius* | *Esox lucius* | *Esox lucius* |
| *Fundulus heteroclitus* | *Fundulus heteroclitus* | *Fundulus heteroclitus* |
| *Haplochromis burtoni* | *Haplochromis burtoni* | *Haplochromis burtoni* |
| *Hippocampus comes* | *Hippocampus comes* | *Hippocampus comes* |
| *Homo sapiens* | *Homo sapiens* | *Homo sapiens* |
| *Ictalurus punctatus* | *Ictalurus punctatus* | *Ictalurus punctatus* |
| *Labrus bergylta* | *Kryptolebias marmoratus* | *Kryptolebias marmoratus* |
| *Larimichthys crocea* | *Labrus bergylta* | *Labrus bergylta* |
| *Latimeria chalumnae* | *Larimichthys crocea* | *Larimichthys crocea* |
| *Lepisosteus oculatus* | *Lates calcarifer* | *Lates calcarifer* |
| *Maylandia zebra* | *Latimeria chalumnae* | *Latimeria chalumnae* |
| *Monopterus albus* | *Lepisosteus oculatus* | *Lepisosteus oculatus* |
| *Neolamprologus brichardi* | *Maylandia zebra* | *Maylandia zebra* |
| *Nothobranchius furzeri* | *Monopterus albus* | *Neolamprologus brichardi* |
| *Notothenia coriiceps* | *Neolamprologus brichardi* | *Nothobranchius furzeri* |
| *Oncorhynchus kisutch* | *Notothenia coriiceps* | *Notothenia coriiceps* |
| *Oncorhynchus mykiss* | *Oncorhynchus kisutch* | *Oncorhynchus kisutch* |
| *Oreochromis niloticus* | *Oncorhynchus mykiss* | *Oncorhynchus mykiss* |
| *Oryzias latipes* | *Oreochromis niloticus* | *Oreochromis niloticus* |
| *Paralichthys olivaceus* | *Oryzias latipes* | *Oryzias latipes* |
| *Poecilia Formosa* | *Paralichthys olivaceus* | *Paralichthys olivaceus* |
| *Poecilia latipinna* | *Poecilia Formosa* | *Poecilia Formosa* |
| *Poecilia Mexicana* | *Poecilia latipinna* | *Poecilia latipinna* |
| *Poecilia reticulata* | *Poecilia Mexicana* | *Poecilia Mexicana* |
| *Pundamilia nyererei* | *Poecilia reticulate* | *Poecilia reticulate* |
| *Rattus norvegicus* | *Pundamilia nyererei* | *Pygocentrus nattereri* |
| *Rhincodon typus* | *Pygocentrus nattereri* | *Rhincodon typus* |
| *Salmo salar* | *Rattus norvegicus* | *Salmo salar* |
| *Scleropages formosus* | *Rhincodon typus* | *Seriola dumerili* |
| *Stegastes partitus* | *Salmo salar* | *Stegastes partitus* |
| *Takifugu rubripes* | *Scleropages formosus* | *Takifugu rubripes* |
| *Tetraodon nigroviridis* | *Seriola dumerili* |  |
| *Xiphophorus maculatus* | *Stegastes partitus* |  |

**Table S3**Summary of Subread Analysis of PacBio RS II sequence data generated for *Tenualosa ilisha* genome assembly from 180 SMRT Cells

| **Job Metric** | **Value** |
| --- | --- |
| Adapter Dimers (0-10bp) | 0.01% |
| Short Inserts (11-100bp) | 0.01% |
| Number of Bases | 61,572,549,341 |
| Number of Reads | 4,787,995 |
| N_50_ Read Length (bp) | 21,233 |
| Mean Read Length (bp) | 12,859 |
| Mean Read Score | 0.87 |

**Table S4 Statistics of Illumina short read data generated for *Tenualosa ilisha* after quality filtering of raw reads**

| **S. No.** | **Sample** | **Read Orientation** | **Mean Read Quality (Phred Score)** | **Number of reads** | **% GC** | **% Q < 10** | **% Q < 10 -20** | **% Q < 20 -30** | **% Q > 30** | **Number of bases (Mb)** | **Mean read length (bp)** |
| --- | --- | --- | --- | --- | --- | --- | --- | --- | --- | --- | --- |
| 1 | TIL960_PE_INS_300-350 | R1 | 37.38 | 104702740 | 44.58 | 0.01 | 0.12 | 1.07 | 98.8 | 9901.93 | 94.57 |
|  |  | R2 | 37.16 | 104702740 | 44.45 | 0.02 | 0.19 | 1.41 | 98.38 | 9626.17 | 91.94 |
| 2 | TIL960_INS_500 | R1 | 36.98 | 98783077 | 44.28 | 0.04 | 0.32 | 0.97 | 98.68 | 9431.91 | 95.48 |
|  |  | R2 | 36.8 | 98783077 | 44.21 | 0.01 | 0.47 | 1.43 | 98.09 | 9252.38 | 93.66 |
| 3 | TIL960_PE_INS_600-700 | R1 | 39.03 | 47222017 | 44.8 | 0.0 | 0.45 | 0.42 | 99.13 | 10484.24 | 222.02 |
|  |  | R2 | 38.68 | 47222017 | 44.74 | 0.0 | 0.73 | 0.67 | 98.6 | 10008.24 | 211.94 |
|  | **Total** |  |  | **501415668** |  |  |  |  |  | **58704.87** |  |

**Table S5** Tissue wise statistics of PacBio transcriptome (Isoseq) data generated from five tissues of *Tenualosa ilisha.*

| **Statistics/Tissues** | **Brain** | **Liver** | **Gill** | **Ovary** | **Testes** | **Total** |
| --- | --- | --- | --- | --- | --- | --- |
| Total generated data | 8.30 Gb | 5.99 Gb | 4.82 Gb | 4.21 Gb | 5.80 Gb | 29.12 GB |
| Mean read length | 1684 | 1616 | 1467 | 1059 | 1033 |  |
| Mean read quality | 95.67 | 94.05 | 94.12 | 98.25 | 98.39 |  |
| Mean number of passes | 12 | 14 | 16 | 29 | 32 |  |
| No. of full length reads | 201345 | 194534 | 196686 | 147897 | 192987 | 933449 |
| No. of non-full length reads | 145367 | 120317 | 111820 | 17917 | 26868 | 422289 |
| No. of high quality isoform reads | 57680 | 28351 | 38232 | 17429 | 30696 | 172388 |
| BioSample Accession | SAMN07977428 | SAMN07977427 | SAMN07977429 | SAMN07977431 | SAMN07977430 |  |
| SRA Accession | SRR6417998 to SRR6418008 | SRR6277490 to SRR6277500 | SRR6418035 to SRR6418045 | SRR6433061 to  SRR6433071 | SRR6432945 to  SRR6432955 |  |

**Table S6** Final statistics of *Tenualosa ilisha*. Genome assembly from present study.

|  | **Fina**l As**sembly statistics** | **Final Assembly**  **(After Quiver p**o**lishing)** |
| --- | --- | --- |
| **Assemblly Size (Mb)** | **762.624459** | **763.185777** |
| Length_cutoff (bp) | 8000 |  |
| Length_cutoff_pr (bp) | 7000 |  |
| Total Contigs | **3052** | ***2867***** |
| Largest Contig | **14.497 Mb** | **17.431Mb** |
| N50 | **2.650 Mb** | 2.624Mb |
| L50 | 85 | 83 |
| N75 | 799.646Kb | 808.693Kb |
| L75 | 212 | 206 |
| # Contigs > 500bp | 3041 | 2866 |
|  | T=8 |  |
|  | Max coverage=100 |  |
|  | Max differ=100 |  |
|  | Min identity= 0.80 |  |
| Total BUSCO groups searched | 4584 | 4584**l** (92.80%) |
| Complete BUSCOs (C) | 3926 | 4256 (87.80% |
| Complete and single-copy BUSCOs (S) | 3689 | 4026 (5.00%) |
| Complete and duplicated BUSCOs (D) | 237 | 230 (3.60%) |
| Fragmented BUSCOs (F) | 344 | 166 (3.60%) |
| Missing BUSCOs (M) | 314 | 162 (3.60%) |

**** Final assembly contains 2864 contigs (762.5 Mb size) after removing mitochondrial (2) and vector (1) sequences.**

**Table S7** Cumulative mapping results of illumina reads (300-350 bp, 500 bp and 600-700 bp) on PacBio assembled draft genome of *Tenualosa ilisha*

| **Characteristics** | **Count** | **Percentage of reads** |
| --- | --- | --- |
| References | 2,864 | - |
| Mapped reads | 572,214,752 | 98.85% |
| Not mapped reads | 6,640,062 | 1.15% |
| Reads in pairs | 536,041,934 | 92.60% |
| Broken paired reads | 36,172,818 | 6.25% |
| Total reads | 578,854,814 | 100.00% |

**Table S8** Benchmarking Universal Single-Copy Orthologs (BUSCO) analysis to assess the completeness of *Tenualosa ilisha* draft genome against highly conserved core genes datasets of Actinopterygii, Eukaryota and Vertebrata lineage.

| **BUSCO Class/Groups** | **Against Vertebrate** | | **Against Actinopterygii** | | **Against Eukaryota** | |
| --- | --- | --- | --- | --- | --- | --- |
|  | **Number of**  **BUSCO**  **Groups** | **% of BUSCO Groups Classified** | **Number of**  **BUSCO**  **Groups** | **% of BUSCO Groups Classified** | **Number of**  **BUSCO**  **Groups** | **% of BUSCO Groups Classified** |
| Total BUSCO groups searched | 2586 |  | 4584 |  | 303 |  |
| Complete BUSCOs (C) | 2397 | 92.70% | 4206 | 91.80% | 282 | 93% |
| - Complete and single-copy BUSCOs (S) | 2292 | 88.60% | 3965 | 86.50% | 268 | 88.40% |
| - Complete and duplicated BUSCOs (D) | 105 | 4.10% | 241 | 5.30% | 14 | 4.60% |
| Fragmented BUSCOs (F) | 127 | 4.90% | 210 | 4.60% | 1 | 0.30% |
| Missing BUSCOs (M) | 62 | 2.40% | 168 | 3.60% | 20 | 6.70% |

**Table 9** Identification of repeat elements in *Tenualosa ilisha draft* genome.

| **Software used** | **Types of repeats** | **Number** | **Size_bp (MB)** | **% of Draft**  **Assembly** |
| --- | --- | --- | --- | --- |
| **Repeat Scout** | Rrepeat elements | 17,280 | 8057714  (8.06 Mbp) | 1.05 |
| **LTR harvester** | LTR elements | 11,977 | 11232420  (11.23 Mbp) | 1.47 |
| **TE class** | Transposable elements | 15, 860 | 7512761  (7.51 Mbp) | 0.98 |
| **TransposonPSI** | Transposon superfamily | 10,698 | 5660628  (5.66 Mbp) | 0.74 |
| **MITE Digger** | Miniature inverted-repeat transposable elements | 132 | 41811  (0.04 Mbp) | 0.005 |
| **MISA** | Simple Sequence Repeats (SSR’s) | 17,85,618 | 64006510  (64.0 Mbp) | 8.39 |
| **tRNAScan-SE** | tRNA’s | 8662 | 2188560  (2.188 Mbp) | 0.29 |
|  | Total |  | 98700404  (98.688Mbp) | 12.925 |

**Table S10 Identification of simple sequence repeats (SSRs) in *Tenualosa ilisha* draft genome assembly**

| **Category** | **Numbers** |
| --- | --- |
| Total number of sequences examined | 2864 |
| Total size of examined sequences (bp) | 762620568 |
| Total number of identified SSRs | 1785618 |
| Number of SSR containing sequences | 2196 |
| Number of sequences containing more than 1 SSR | 2038 |
| Number of SSRs present in compound formation | 911642 |
| Total sequence length of SSRs (bp) | 3558431 |
| Average Relative abundance (No. of SSRs/Mb) | 389.73 |
| Average Relative density (Sequence length of SSRs (bp/Mb) | 776.67 |

**Table S11** Transposable elements identified in *Tenoalosa ilisha* draft genome assembly

| **S.No** | **Types** | **Number** |
| --- | --- | --- |
|  | DNA | 4132 |
|  | LINE | 3248 |
|  | SINE | 780 |
|  | LTR | 3594 |
|  | Non-LTR | 246 |
|  | Retro | 2874 |
|  | Unclear | 986 |
|  | **TOTAL** | **15860** |

**Table S12 Super families of Transposable elements classified in *Tenoalosa ilisha* draft genome assembly**

| **S.No** | **Superfamilies** | **Number** |
| --- | --- | --- |
|  | Ty1Copia | 1128 |
|  | Gypsy | 3898 |
|  | Crypton | 9 |
|  | HelitronORF | 5 |
|  | Mariner | 1271 |
|  | Cacta | 1675 |
|  | DDE-1 | 52 |
|  | hAT | 330 |
|  | Itr_Roo | 1099 |
|  | MUDR_A_B | 54 |
|  | P_element | 6 |
|  | Piggybac | 597 |
|  | ISC1316 | 34 |
|  | Mariner_ant | 540 |
|  | **TOTAL** | **10,698** |

**Table S13** The tRNA genes identified in *Tenoalosa ilisha* draft genome assembly along with their respective anticodons

| **Amino acids** | **Anticodons (Frequency)** | | | | | | **Total** |
| --- | --- | --- | --- | --- | --- | --- | --- |
| Ala | AGC: 83 | GGC: 1 | CGC: 19 | TGC: 62 |  |  | 165 |
| Gly | ACC: 5 | GCC: 120 | CCC: 44 | TCC: 99 |  |  | 268 |
| Pro | AGG: 48 | GGG: 0 | CGG: 36 | TGG: 46 |  |  | 130 |
| Thr | AGT: 111 | GGT: 0 | CGT: 17 | TGT: 49 |  |  | 177 |
| Val | AAC: 37 | GAC: 0 | CAC: 27 | TAC: 30 |  |  | 98 |
| Ser | AGA: 27 | GGA: 0 | CGA: 32 | TGA: 36 | ACT: 1 | GCT: 89 | 185 |
| Arg | ACG: 42 | GCG: 0 | CCG: 5 | TCG: 96 | CCT: 178 | TCT: 45 | 366 |
| Leu | AAG: 135 | GAG: 0 | CAG: 36 | TAG: 99 | CAA: 28 | TAA: 16 | 314 |
| Phe | AAA: 0 | GAA: 26 |  |  |  |  | 26 |
| Asn | ATT:1 | GTT: 69 |  |  |  |  | 70 |
| Lys |  |  | CTT: 65 | TTT: 152 |  |  | 217 |
| Asp | ATC: 0 | GTC: 87 |  |  |  |  | 87 |
| Glu |  |  | CTC: 51 | TTC: 38 |  |  | 89 |
| His | ATG: 0 | GTG: 43 |  |  |  |  | 43 |
| Gln |  |  | CTG: 25 | TTG: 30 |  |  | 55 |
| Ile | AAT: 48 | GAT: 1 |  | TAT: 19 |  |  | 68 |
| Met |  |  | CAT: 301 |  |  |  | 301 |
| Tyr | ATA: 0 | GTA: 44 |  |  |  |  | 44 |
| Cys | ACA: 0 | GCA: 31 |  |  |  |  | 31 |
| Trp |  |  | CCA: 67 |  |  |  | 67 |
| SelCys |  |  |  | TCA: 4 |  |  | 4 |
| Supres |  |  | CTA: 1 | TTA: 0 |  |  | 1 |
| Pseudogenes | AGC: 83 | GGC: 1 | CGC: 19 | TGC: 62 |  |  | 5802 |
| Unknown isotypes | ACC: 5 | GCC: 120 | CCC: 44 | TCC: 99 |  |  | 45 |
| **Total tRNA** | | | | | | | **8653** |

**Table S14** Benchmarking Universal Single-Copy Orthologs (BUSCO) analysis of potentially predicted gene sets of *Tenualosa ilisha* to assess the completeness in comparison to highly conserved core genes of Actinopterygii

| **BUSCO Class** | **Number of Genes** | **% of Total Actinopterigii BUSCO Group**  **(n: 4584)** |
| --- | --- | --- |
| Complete BUSCOs (C) | 3992 | 87.10 % |
| - Complete and single-copy BUSCOs (S) | 3691 | 80.50 % |
| - Complete and duplicated BUSCOs (D) | 301 | 6.60 % |
| Fragmented BUSCOs (F) | 401 | 8.70 % |
| Missing BUSCOs (M) | 191 | 4.20 % |

**Table S15** Summary of gene annotations in *Tenualosa ilisha*, by comparing with different public databases.

| **Summary** | **Number** |
| --- | --- |
| Total number of predicted coding genes | 33042 |
| - Annotated genes against Nr/Swissprot database | 31937 |
| - Uncharacterized and unidentified protein family | 347 |
| - Pseudogenes | 18 |
| - Unannotated genes | 740 |
|  |  |
| - with GO ID | 32766 |
| - with InterPro IDs | 28559 |
| PFAM | 26582 |
| SMART | 14792 |
| PRINT | 7365 |
| PRODOM | 352 |
| Number of genes with KEGG accession | 21814 |
|  |  |
| Transposable elements | 832 |
| Retroviruses | 341 |
| Transposons | 221 |

**Table S16 . 61 Gene Ontology terms pertaining to homeostasis** in Hilsa, under which a total of 512 genes identified.

| **S No** | **GO_ID** | **GO_Description** | **No of Genes** |
| --- | --- | --- | --- |
|  | **GO:0006874** | cellular calcium ion homeostasis | 63 |
|  | **GO:0001894** | tissue homeostasis | 51 |
|  | **GO:0006879** | cellular iron ion homeostasis | 48 |
|  | **GO:0055072** | iron ion homeostasis | 34 |
|  | **GO:0003091** | renal water homeostasis | 27 |
|  | **GO:0006883** | cellular sodium ion homeostasis | 25 |
|  | **GO:0009414** | response to water deprivation | 25 |
|  | **GO:0006882** | cellular zinc ion homeostasis | 24 |
|  | **GO:0055074** | calcium ion homeostasis | 23 |
|  | **GO:0010960** | magnesium ion homeostasis | 19 |
|  | **GO:0030007** | cellular potassium ion homeostasis | 17 |
|  | **GO:0032469** | endoplasmic reticulum calcium ion homeostasis | 17 |
|  | **GO:0030644** | cellular chloride ion homeostasis | 16 |
|  | **GO:0019725** | cellular homeostasis | 15 |
|  | **GO:0009992** | cellular water homeostasis | 15 |
|  | **GO:0048871** | multicellular organismal homeostasis | 14 |
|  | **GO:0006873** | cellular ion homeostasis | 13 |
|  | **GO:0055075** | potassium ion homeostasis | 13 |
|  | **GO:0006884** | cell volume homeostasis | 12 |
|  | **GO:0006878** | cellular copper ion homeostasis | 11 |
|  | **GO:0055065** | metal ion homeostasis | 11 |
|  | **GO:0070295** | renal water absorption | 11 |
|  | **GO:0030643** | cellular phosphate ion homeostasis | 10 |
|  | **GO:0055062** | phosphate ion homeostasis | 10 |
|  | **GO:0033561** | regulation of water loss via skin | 10 |
|  | **GO:0015250** | water channel activity | 10 |
|  | **GO:0051560** | mitochondrial calcium ion homeostasis | 9 |
|  | **GO:0060586** | multicellular organismal iron ion homeostasis | 9 |
|  | **GO:0006833** | water transport | 9 |
|  | **GO:0050891** | multicellular organismal water homeostasis | 8 |
|  | **GO:0010961** | cellular magnesium ion homeostasis | 7 |
|  | **GO:0098771** | inorganic ion homeostasis | 7 |
|  | **GO:0055069** | zinc ion homeostasis | 7 |
|  | **GO:0050801** | ion homeostasis | 6 |
|  | **GO:0006111** | regulation of gluconeogenesis | 6 |
|  | **GO:0042631** | cellular response to water deprivation | 4 |
|  | **GO:0030641** | regulation of cellular pH | 4 |
|  | **GO:0097274** | urea homeostasis | 4 |
|  | **GO:0003097** | renal water transport | 3 |
|  | **GO:0055078** | sodium ion homeostasis | 3 |
|  | **GO:0035377** | Trans-epithelial water transport | 3 |
|  | **GO:0030104** | water homeostasis | 3 |
|  | **GO:0005372** | water trans-membrane transporter activity | 3 |
|  | **GO:0055081** | anion homeostasis | 2 |
|  | **GO:0055080** | cation homeostasis | 2 |
|  | **GO:0030320** | cellular monovalent inorganic anion homeostasis | 2 |
|  | **GO:0046916** | cellular transition metal ion homeostasis | 2 |
|  | **GO:0097277** | cellular urea homeostasis | 2 |
|  | **GO:2000070** | regulation of response to water deprivation | 2 |
|  | **GO:0051563** | smooth endoplasmic reticulum calcium ion homeostasis | 2 |
|  | **GO:1903118** | urate homeostasis | 2 |
|  | **GO:0030002** | cellular anion homeostasis | 1 |
|  | **GO:0006876** | cellular cadmium ion homeostasis | 1 |
|  | **GO:0030026** | cellular manganese ion homeostasis | 1 |
|  | **GO:0006875** | cellular metal ion homeostasis | 1 |
|  | **GO:0055068** | cobalt ion homeostasis | 1 |
|  | **GO:0055070** | copper ion homeostasis | 1 |
|  | **GO:0072507** | divalent inorganic cation homeostasis | 1 |
|  | **GO:0032468** | Golgi calcium ion homeostasis | 1 |
|  | **GO:0050824** | obsolete water binding | 1 |
|  | **GO:1990785** | response to water-immersion restraint stress | 1 |

**Table S17: Genes (512) identified under 61 GO terms related to homeostasis in *Tenualosa ilisha***

| **SeqName** | **Gene Symbol** | **Description** | **e-Value** | **#GO** |
| --- | --- | --- | --- | --- |
| g52114.t1 | OTO1A | Otolin-1-A | 1.62E-180 | 19 |
| g59338.t1 | CPSM | Carbamoyl-phosphate synthase | 0 | 53 |
| g30355.t1 | VATD | V-type proton ATPase subunit D | 0 | 41 |
| g14365.t1 | VATB2 | V-type proton ATPase subunit brain isoform | 0 | 41 |
| g21099.t1 | TNF10 | Tumor necrosis factor ligand superfamily member 10 | 4.44E-33 | 35 |
| g463.t1 | ANXA1 | Annexin A1 | 2.37E-150 | 71 |
| g6205.t1 | VGL2A | Vesicular glutamate transporter | 0 | 66 |
| g1138.t1 | VGFR1 | Vascular endothelial growth factor receptor 1 | 0 | 65 |
| g51118.t1 | GNAS | Guanine nucleotide-binding G(s) subunit alpha | 4.71E-98 | 69 |
| g24984.t1 | AP2B1 | AP-2 complex subunit beta | 0 | 85 |
| g41771.t1 | AP2A1 | AP-2 complex subunit alpha-1 | 0 | 97 |
| g50028.t1 | VGFR4 | Vascular endothelial growth factor receptor kdr-like | 0 | 64 |
| g62148.t1 | VGFR2 | Vascular endothelial growth factor receptor 2 | 0 | 66 |
| g173.t1 | TIF1A | Transcription intermediary factor 1-alpha | 2.98E-23 | 69 |
| g16993.t1 | TRI35 | Tripartite motif-containing 35 | 0 | 66 |
| g19069.t1 | TRI67 | Tripartite motif-containing 67 | 2.03E-86 | 48 |
| g35883.t1 | TIF1B | Transcription intermediary factor 1-beta | 6.57E-57 | 52 |
| g24257.t1 | DMP4 | Extracellular serine threonine kinase FAM20C | 5.91E-87 | 37 |
| g43248.t1 | CP27B | 25-hydroxyvitamin D-1 alpha mitochondrial | 3.77E-158 | 27 |
| g34043.t1 | C2AIL | CDKN2AIP N-terminal | 0 | 27 |
| g34050.t1 | CP27A | Sterol 26- mitochondrial | 4.96E-151 | 26 |
| g11059.t1 | CP2A5 | Cytochrome P450 2A5 | 8.50E-179 | 32 |
| g33006.t1 | KMCP1 | Kidney mitochondrial carrier 1 | 0 | 19 |
| g44240.t1 | KLOTB | Beta-klotho | 0 | 23 |
| g45048.t1 | ANXA5 | Annexin A5 | 5.13E-155 | 38 |
| g32805.t1 | GPR83 | Probable G- coupled receptor 83 | 1.44E-61 | 21 |
| g60074.t1 | E2F5 | Transcription factor E2F5 | 4.12E-153 | 31 |
| g23739.t1 | E2F4 | Transcription factor E2F4 | 1.11E-155 | 27 |
| g17556.t1 | TRPV4 | Transient receptor potential cation channel subfamily V member 4 | 1.34E-67 | 92 |
| g5028.t1 | TRPV2 | Transient receptor potential cation channel subfamily V member 2 | 1.04E-100 | 100 |
| g57196.t1 | TRPV5 | Transient receptor potential cation channel subfamily V member 5 | 0 | 99 |
| g7781.t1 | CLCN7 | H(+) Cl(-) exchange transporter 7 | 0 | 13 |
| g48180.t1 | S12A7 | Solute carrier family 12 member 7 | 0 | 16 |
| g52256.t1 | S12A5 | Solute carrier family 12 member 5 | 0 | 28 |
| g23971.t1 | S12A9 | Solute carrier family 12 member 9 | 0 | 27 |
| g39976.t1 | S12A6 | Solute carrier family 12 member 6 | 0 | 27 |
| g29727.t1 | NPM | Nucleoplasmin | 3.18E-68 | 64 |
| g18179.t1 | NPRL2 | GATOR complex NPRL2 | 1.80E-64 | 60 |
| g19360.t1 | CLD10 | Claudin-10 | 0 | 15 |
| g11057.t1 | NRAP | Nebulin-related-anchoring | 0 | 96 |
| g16455.t1 | ITA3 | Integrin alpha-3 | 9.69E-170 | 44 |
| g44823.t1 | NMDZ1 | Glutamate receptor NMDA 1 | 2.27E-173 | 66 |
| g44824.t1 | NMI | N-myc-interactor | 0 | 70 |
| g45111.t1 | CSTN2 | Calsyntenin-2 | 0 | 24 |
| g58846.t1 | GPR98 | G- coupled receptor 98 | 0 | 32 |
| g34362.t1 | NALCN | Sodium leak channel non-selective | 0 | 21 |
| g7797.t1 | GLRX | Glutaredoxin | 1.23E-166 | 26 |
| g61920.t1 | GLRK | Probable glutamate receptor | 1.11E-165 | 26 |
| g24019.t1 | GRIK4 | Glutamate receptor kainate 4 | 0 | 36 |
| g2851.t1 | GRIK5 | Glutamate receptor kainate 5 | 0 | 34 |
| g22513.t1 | GRIK2 | Glutamate receptor kainate 2 | 2.77E-170 | 43 |
| g4782.t1 | GRIK1 | Glutamate receptor kainate 1 | 6.36E-154 | 34 |
| g7847.t1 | SYPM | Probable proline--tRNA mitochondrial | 4.38E-73 | 26 |
| g50343.t1 | CRFR1 | Corticotropin-releasing factor receptor 1 | 1.07E-88 | 55 |
| g56071.t1 | ACHA7 | Neuronal acetylcholine receptor subunit alpha-7 Flags | 0 | 50 |
| g24950.t1 | GPC6A | G- coupled receptor family C group 6 member A | 0 | 11 |
| g9294.t1 | NCKX4 | Sodium potassium calcium exchanger 4 | 0 | 17 |
| g4869.t1 | NCKX3 | Sodium potassium calcium exchanger 3 | 0 | 15 |
| g45761.t1 | NCKX5 | Sodium potassium calcium exchanger 5 | 0 | 18 |
| g9457.t1 | PYGM | Glycogen muscle form | 0 | 22 |
| g46875.t1 | PYGO1 | Pygopus homolog 1 | 0 | 16 |
| g51297.t1 | PYGL | Glycogen liver form | 0 | 16 |
| g27039.t1 | STIM1 | Stromal interaction molecule 1 Flags | 0 | 26 |
| g914.t1 | STIM2 | Stromal interaction molecule 2 Flags | 7.66E-138 | 26 |
| g9796.t1 | TGM5 | -glutamine gamma-glutamyltransferase 5 | 8.18E-136 | 13 |
| g37219.t1 | ITA6 | Integrin alpha-6 | 0 | 47 |
| g26024.t1 | SV2B | Synaptic vesicle glyco 2B | 7.61E-61 | 10 |
| g52618.t1 | SSR1 | Somatostatin receptor type 1 | 2.21E-12 | 29 |
| g63766.t1 | NCKX2 | Sodium potassium calcium exchanger 2 | 0 | 18 |
| g37206.t1 | VEGFC | Vascular endothelial growth factor C | 0 | 44 |
| g14498.t1 | MRCKA | Serine threonine- kinase MRCK alpha | 2.13E-89 | 38 |
| g51960.t1 | CASR | Extracellular calcium-sensing receptor | 0 | 33 |
| g25987.t1 | CCL11 | Eotaxin | 1.09E-06 | 23 |
| g59987.t1 | PTK6 | -tyrosine kinase 6 | 1.10E-12 | 17 |
| g14965.t1 | ANX13 | Annexin A13 | 8.57E-117 | 43 |
| g40257.t1 | HEXDC | Hexosaminidase D | 0 | 37 |
| g40256.t1 | HEXB | Beta-hexosaminidase subunit beta | 5.94E-61 | 37 |
| g63776.t1 | HEXA | Beta-hexosaminidase subunit alpha | 1.92E-54 | 37 |
| g7817.t1 | TNR5 | Tumor necrosis factor receptor superfamily member 5 | 3.51E-23 | 22 |
| g9151.t1 | ZNT1 | Zinc transporter 1 | 2.80E-157 | 19 |
| g10679.t1 | PK3CB | Phosphatidylinositol 4,5-bisphosphate 3-kinase catalytic subunit beta isoform | 0 | 61 |
| g2986.t1 | PK3CA | Phosphatidylinositol 4,5-bisphosphate 3-kinase catalytic subunit alpha isoform | 0 | 61 |
| g15809.t1 | PK3CD | Phosphatidylinositol 4,5-bisphosphate 3-kinase catalytic subunit delta isoform | 0 | 67 |
| g58523.t1 | PK3CG | Phosphatidylinositol 4,5-bisphosphate 3-kinase catalytic subunit gamma isoform | 0 | 66 |
| g63597.t1 | WWP1 | NEDD4-like E3 ubiquitin- ligase WWP1 | 4.30E-106 | 55 |
| g1002.t1 | KPCB | kinase C beta type | 4.44E-31 | 85 |
| g38948.t1 | KPCA | kinase C alpha type | 0 | 87 |
| g8612.t1 | PTHB1 | PTHB1 | 5.72E-122 | 20 |
| g22795.t1 | ACHA9 | Neuronal acetylcholine receptor subunit alpha-9 | 5.14E-78 | 42 |
| g3164.t1 | KPCD | kinase C delta type | 1.04E-21 | 65 |
| g57406.t1 | STC | Stanniocalcin | 8.38E-106 | 33 |
| g41766.t1 | GCN1 | eIF-2-alpha kinase activator GCN1 | 7.70E-142 | 31 |
| g45622.t1 | CCL4 | C-C motif chemokine 4 homolog | 8.27E-15 | 36 |
| g44124.t1 | STC2 | Stanniocalcin-2 | 3.56E-50 | 22 |
| g5612.t1 | B2CL1 | Bcl-2 1 | 1.59E-79 | 78 |
| g15207.t1 | AT133 | Probable cation-transporting ATPase 13A3 | 3.86E-173 | 22 |
| g33816.t1 | ZNT10 | Zinc transporter 10 | 6.59E-161 | 17 |
| g46149.t1 | TGFB2 | Transforming growth factor beta-2 | 6.57E-118 | 78 |
| g13541.t1 | AT2A1 | Sarcoplasmic endoplasmic reticulum calcium ATPase 1 | 1.67E-166 | 44 |
| g62531.t1 | AT1B2 | Sodium potassium-transporting ATPase subunit beta-2 | 3.15E-158 | 39 |
| g61201.t1 | STMN1 | Stathmin | 1.81E-177 | 27 |
| g49978.t1 | PTHY | Parathyroid hormone | 1.54E-09 | 30 |
| g1580.t1 | AT132 | Cation-transporting ATPase 13A2 | 0 | 37 |
| g6561.t1 | KCRT | Creatine testis isozyme | 0 | 16 |
| g48780.t1 | KCRM | Creatine kinase M-type | 0 | 16 |
| g44809.t1 | CAC1B | Voltage-dependent N-type calcium channel subunit alpha-1B | 0 | 57 |
| g52443.t1 | CAC1A | Voltage-dependent P Q-type calcium channel subunit alpha-1A | 0 | 56 |
| g51315.t1 | MRP2 | Canalicular multispecific organic anion transporter 1 | 0 | 43 |
| g63477.t1 | WNK2 | Serine threonine- kinase WNK2 | 0 | 32 |
| g7999.t1 | WNK3 | Serine threonine- kinase WNK3 | 0 | 36 |
| g16494.t1 | WNK4 | Serine threonine- kinase WNK4 | 3.70E-104 | 37 |
| g12690.t1 | WNK1 | Serine threonine- kinase WNK1 | 1.40E-172 | 37 |
| g46252.t1 | FAK1 | Focal adhesion kinase 1 | 0 | 133 |
| g39861.t1 | FAK2 | -tyrosine kinase 2-beta | 9.08E-106 | 129 |
| g15030.t1 | CAC1E | Voltage-dependent R-type calcium channel subunit alpha-1E | 0 | 52 |
| g6127.t1 | TM9S2 | Transmembrane 9 superfamily member 2 Flags | 0 | 11 |
| g61009.t1 | COPT2 | Probable low affinity copper uptake 2 | 1.49E-91 | 12 |
| g31124.t1 | A4 | Amyloid beta A4 | 0 | 85 |
| g55812.t1 | ATP23 | Mitochondrial inner membrane protease ATP23 homolog | 5.51E-29 | 17 |
| g21010.t1 | APMAP | Adipocyte plasma membrane-associated | 0 | 61 |
| g55983.t1 | ATP7A | Copper-transporting ATPase 1 | 4.59E-51 | 50 |
| g55984.t1 | ATP7B | Copper-transporting ATPase 2 | 0 | 77 |
| g27503.t1 | ATP9A | Probable phospholipid-transporting ATPase IIA | 0 | 74 |
| g13477.t1 | TM9S3 | Transmembrane 9 superfamily member 3 | 0 | 12 |
| g59142.t1 | A6108 | Uncharacterized secreted ARB | 0 | 80 |
| g1762.t1 | AQP4 | Aquaporin-4 | 3.33E-40 | 31 |
| g60964.t1 | CXD2 | Gap junction delta-2 | 5.55E-21 | 27 |
| g43783.t1 | SLP1 | Shematrin 1 | 7.02E-25 | 29 |
| g17032.t1 | DEDD2 | DNA-binding death effector domain-containing 2 | 1.78E-168 | 16 |
| g5440.t1 | FBX40 | F-box only 40 | 1.28E-103 | 16 |
| g38029.t1 | MCL1A | L-Myc-1a | 3.44E-48 | 35 |
| g17338.t1 | MCL1 | Induced myeloid leukemia cell differentiation Mcl-1 homolog | 7.11E-39 | 29 |
| g30120.t1 | TENS3 | Tensin-3 | 3.81E-157 | 21 |
| g31983.t1 | TENS4 | Tensin-4 Flags | 0 | 17 |
| g13777.t1 | TENS | Tensin | 0 | 17 |
| g59373.t1 | TOB1 | Tob1 | 7.67E-07 | 10 |
| g37203.t1 | TNS2 | Tensin-2 | 1.38E-08 | 10 |
| g59380.t1 | TENS1 | Tensin-1 | 0 | 14 |
| g55672.t1 | TENX | Tenascin-X | 5.78E-103 | 12 |
| g59305.t1 | ABCB6 | ATP-binding cassette sub-family B member mitochondrial | 0 | 44 |
| g49373.t1 | RHCG1 | Ammonium transporter Rh type C 1 | 0 | 16 |
| g17321.t1 | RHBGA | Ammonium transporter Rh type B-A | 0 | 14 |
| g40.t1 | RHCG | Ammonium transporter Rh type C | 0 | 13 |
| g63695.t1 | RHDF1 | Inactive rhomboid 1 | 0 | 15 |
| g47476.t1 | APOD | Apolipo D | 2.90E-60 | 38 |
| g46258.t1 | GPR22 | Probable G- coupled receptor 22 | 3.01E-89 | 27 |
| g19152.t1 | RHAG | Ammonium transporter Rh type A | 2.19E-93 | 11 |
| g2050.t1 | B3AT | Band 3 anion exchange | 0 | 18 |
| g58105.t1 | MRP5 | Multidrug resistance-associated 5 | 0 | 22 |
| g45974.t1 | P2RY4 | P2Y purinoceptor 4 | 1.09E-141 | 16 |
| g48088.t1 | PP1R7 | phosphatase 1 regulatory subunit 7 | 0 | 88 |
| g45677.t1 | RHBD2 | Rhomboid domain-containing 2 | 0 | 19 |
| g35404.t1 | SMAD4 | Mothers against decapentaplegic homolog 4 | 8.76E-34 | 107 |
| g6460.t1 | PRSS8 | Prostasin | 1.60E-25 | 21 |
| g14597.t1 | TMPS6 | Transmembrane protease serine 6 | 2.22E-36 | 19 |
| g45328.t1 | HPLN1 | Hyaluronan and proteoglycan link 1 | 0 | 15 |
| g10290.t1 | CERU | Ceruloplasmin | 3.13E-172 | 14 |
| g58562.t1 | CETA | 2-epi-5-epi-valiolone synthase | 0 | 14 |
| g8119.t1 | S39A1 | Zinc transporter ZIP1 | 2.05E-93 | 17 |
| g5842.t1 | S39A3 | Zinc transporter ZIP3 | 5.36E-131 | 17 |
| g12432.t1 | PCFT | Proton-coupled folate transporter | 0 | 17 |
| g40013.t1 | HEM0 | 5-aminolevulinate erythroid- mitochondrial | 7.43E-68 | 16 |
| g40014.t1 | HEM1 | 5-aminolevulinate mitochondrial | 0 | 14 |
| g56099.t1 | ABCB8 | ATP-binding cassette sub-family B member mitochondrial Flags | 0 | 19 |
| g47481.t1 | NADL2 | Inactive N-acetylated-alpha-linked acidic dipeptidase 2 | 0 | 13 |
| g19404.t1 | ALRF2 | Aly REF export factor 2 | 1.30E-70 | 18 |
| g39150.t1 | ISK1 | Probable pancreatic secretory ase inhibitor | 1.35E-84 | 16 |
| g9539.t1 | ABCG2 | ATP-binding cassette sub-family G member 2 | 0 | 22 |
| g3498.t1 | TMPS9 | Transmembrane protease serine 9 | 8.84E-49 | 12 |
| g27915.t1 | HEPS | Serine protease hepsin Contains | 1.80E-22 | 9 |
| g15701.t1 | FRIH | heavy subunit | 1.62E-69 | 5 |
| g57096.t1 | SMAD5 | Mothers against decapentaplegic homolog 5 | 0 | 97 |
| g36707.t1 | ACOD | Acyl- desaturase | 4.59E-70 | 32 |
| g32856.t1 | S40A1 | Solute carrier family 40 member 1 | 0 | 26 |
| g63355.t1 | IRF1 | Interferon regulatory factor 1 | 0 | 22 |
| g36705.t1 | ACOC | Cytoplasmic aconitate hydratase | 0 | 22 |
| g50554.t1 | SCAR5 | Scavenger receptor class A member 5 | 0 | 21 |
| g8294.t1 | FMC1 | FMC1 homolog | 0 | 19 |
| g37735.t1 | ABCB7 | ATP-binding cassette sub-family B member mitochondrial | 0 | 27 |
| g56990.t1 | FREM1 | FRAS1-related extracellular matrix 1 | 1.88E-57 | 39 |
| g7040.t1 | HIF1A | Hypoxia-inducible factor 1-alpha | 4.34E-148 | 98 |
| g29519.t1 | EPB1B | Ephrin type-B receptor 1-B | 0 | 99 |
| g19492.t1 | HMR1 | Major histocompatibility complex class I-related gene | 3.53E-131 | 54 |
| g52968.t1 | HMOX | Heme oxygenase | 1.49E-110 | 56 |
| g53743.t1 | HMOX2 | Heme oxygenase 2 | 3.06E-119 | 45 |
| g4026.t1 | EGR1 | Early growth response 1 | 2.30E-118 | 34 |
| g11927.t1 | EGLN3 | Egl nine homolog 3 | 7.73E-79 | 34 |
| g55484.t1 | EGLN1 | Egl nine homolog 1 | 2.37E-80 | 34 |
| g22527.t1 | SIM2 | Single-minded homolog 2 | 0 | 67 |
| g54579.t1 | SIM24 | Small integral membrane 24 Flags | 9.28E-136 | 64 |
| g32311.t1 | ABCG4 | ATP-binding cassette sub-family G member 4 | 2.24E-79 | 26 |
| g4775.t1 | SODE | Extracellular superoxide dismutase | 2.46E-90 | 66 |
| g39661.t1 | S41A1 | Solute carrier family 41 member 1 | 0 | 23 |
| g14599.t1 | TMPS7 | Transmembrane protease serine 7 | 3.77E-34 | 17 |
| g64472.t1 | ADIPO | Adiponectin | 7.74E-15 | 15 |
| g63390.t1 | S41A3 | Solute carrier family 41 member 3 | 0 | 9 |
| g21419.t1 | S41A2 | Solute carrier family 41 member 2 | 1.88E-128 | 9 |
| g54391.t1 | S43A3 | Solute carrier family 43 member 3 | 5.66E-176 | 8 |
| g11179.t1 | EDNRA | Endothelin-1 receptor | 5.78E-37 | 56 |
| g14039.t1 | TRPM7 | Transient receptor potential cation channel subfamily M member 7 | 0 | 35 |
| g14016.t1 | TRPM3 | Transient receptor potential cation channel subfamily M member 3 | 0 | 36 |
| g8071.t1 | AT2L1 | Ethanolamine-phosphate phospho-lyase | 0 | 26 |
| g33796.t1 | ENPP2 | Ectonucleotide pyrophosphatase phosphodiesterase family member 2 | 0 | 32 |
| g49284.t1 | GPD1L | Glycerol-3-phosphate dehydrogenase 1 | 0 | 7 |
| g19692.t1 | XPR1 | Xenotropic and polytropic retrovirus receptor 1 | 0 | 14 |
| g912.t1 | NPT2B | Sodium-dependent phosphate transport 2B | 0 | 29 |
| g30295.t1 | NPTN | Neuroplastin | 0 | 41 |
| g46985.t1 | NPT2A | Sodium-dependent phosphate transport 2A | 0 | 41 |
| g33958.t1 | NHRF2 | Na(+) H(+) exchange regulatory cofactor NHE-RF2 | 1.42E-55 | 33 |
| g29821.t1 | NHRF3 | Na(+) H(+) exchange regulatory cofactor NHE-RF3 | 1.58E-29 | 65 |
| g14585.t1 | NHRF1 | Na(+) H(+) exchange regulatory cofactor NHE-RF1 | 5.87E-73 | 55 |
| g34760.t1 | DRD3 | D(3) dopamine receptor | 2.53E-43 | 100 |
| g5132.t1 | DRD2L | D(2)-like dopamine receptor | 0 | 99 |
| g42971.t1 | KCC2G | Calcium calmodulin-dependent kinase type II subunit gamma | 1.02E-32 | 57 |
| g894.t1 | KCA10 | Potassium voltage-gated channel subfamily A member 10 | 0 | 70 |
| g2921.t1 | KC2D1 | Calcium calmodulin-dependent kinase type II delta 1 chain | 1.42E-90 | 70 |
| g5319.t1 | KCC2B | Calcium calmodulin-dependent kinase type II subunit beta | 1.05E-159 | 83 |
| g52780.t1 | KCMA1 | Calcium-activated potassium channel subunit alpha-1 | 2.03E-119 | 16 |
| g41886.t1 | DRD4 | D(4) dopamine receptor | 8.53E-161 | 90 |
| g28544.t1 | KCNJ2 | Inward rectifier potassium channel 2 | 0 | 28 |
| g23342.t1 | KCNJ3 | G -activated inward rectifier potassium channel 1 | 0 | 28 |
| g2972.t1 | AT1B3 | Sodium potassium-transporting ATPase subunit beta-3 | 1.52E-127 | 17 |
| g56481.t1 | AT1B4 | ATP1B4 | 5.73E-119 | 18 |
| g6237.t1 | AT233 | Sodium potassium-transporting ATPase subunit beta-233 | 1.25E-106 | 17 |
| g56265.t1 | ATP5E | ATP synthase subunit mitochondrial | 4.70E-23 | 20 |
| g32806.t1 | CSKP | Peripheral plasma membrane CASK | 1.04E-55 | 99 |
| g30895.t1 | RECQ5 | ATP-dependent DNA helicase Q5 | 1.86E-175 | 19 |
| g7966.t1 | MIP | Lens fiber major intrinsic | 1.81E-151 | 38 |
| g23803.t1 | RED | Red | 6.39E-160 | 31 |
| g57821.t1 | TMPS3 | Transmembrane protease serine 3 | 9.41E-124 | 12 |
| g45286.t1 | TMTC1 | Transmembrane and TPR repeat-containing 1 | 1.07E-111 | 10 |
| g11888.t1 | TMPS4 | Transmembrane protease serine 4 | 3.05E-107 | 11 |
| g17727.t1 | NEDD4 | E3 ubiquitin- ligase NEDD4 | 0 | 96 |
| g11893.t1 | TMPSD | Transmembrane protease serine 13 | 3.97E-102 | 13 |
| g56553.t1 | MCR | Mineralocorticoid receptor | 1.36E-104 | 33 |
| g11171.t1 | SGK3 | Serine threonine- kinase Sgk3 | 3.17E-180 | 43 |
| g7541.t1 | SGK1 | Serine threonine- kinase Sgk1 | 0 | 42 |
| g1106.t1 | SGMR1 | Sigma non-opioid intracellular receptor 1 | 0 | 46 |
| g7149.t1 | SGK2 | Serine threonine- kinase Sgk2 | 0 | 43 |
| g40878.t1 | SL9A2 | Sodium hydrogen exchanger 2 | 0 | 63 |
| g40880.t1 | NHEB | Na(+) H(+) exchanger beta | 2.47E-82 | 63 |
| g60233.t1 | NHEJ1 | Non-homologous end-joining factor 1 | 0 | 58 |
| g56341.t1 | SL9A3 | Sodium hydrogen exchanger 3 | 0 | 46 |
| g28284.t1 | NEDD1 | NEDD1 | 0 | 100 |
| g56554.t1 | MCRI1 | Mapk-regulated corepressor-interacting 1 | 3.76E-17 | 22 |
| g20858.t1 | NAC2 | Sodium calcium exchanger 2 | 0 | 48 |
| g29422.t1 | NAC1 | Sodium calcium exchanger 1 | 0 | 68 |
| g52634.t1 | NAC3 | Sodium calcium exchanger 3 | 0 | 62 |
| g39174.t1 | CCSAP | cilia and spindle-associated | 1.79E-130 | 33 |
| g53058.t1 | AQP8 | Aquaporin-8 | 5.53E-95 | 27 |
| g29152.t1 | AQP11 | Aquaporin-11 | 7.41E-52 | 29 |
| g32015.t1 | AQP7 | Aquaporin-7 | 6.14E-63 | 29 |
| g32081.t1 | AQP1 | Aquaporin-1 | 4.66E-115 | 70 |
| g19621.t1 | AQP10 | Aquaporin-10 | 7.19E-27 | 77 |
| g13035.t1 | S6A14 | Sodium- and chloride-dependent neutral and basic amino acid transporter B(0+) | 4.84E-16 | 16 |
| g12999.t1 | S6A13 | Sodium- and chloride-dependent GABA transporter 2 | 5.10E-149 | 17 |
| g24282.t1 | AQP9 | Aquaporin-9 | 9.24E-41 | 14 |
| g57239.t1 | AQP3 | Aquaporin-3 | 2.49E-144 | 37 |
| g58863.t1 | AQR | Intron-binding aquarius | 5.90E-118 | 37 |
| g55455.t1 | SC6A1 | Sodium- and chloride-dependent GABA transporter 1 | 1.11E-88 | 15 |
| g62647.t1 | S6A12 | Sodium- and chloride-dependent betaine transporter | 0 | 15 |
| g1829.t1 | S39AB | Zinc transporter ZIP11 | 0 | 12 |
| g55917.t1 | S39A9 | Zinc transporter ZIP9 | 5.75E-154 | 10 |
| g12816.t1 | LYOX | -lysine 6-oxidase | 0 | 131 |
| g594.t1 | LYN | Tyrosine- kinase Lyn | 0 | 122 |
| g58456.t1 | HCN1 | Potassium sodium hyperpolarization-activated cyclic nucleotide-gated channel 1 | 0 | 123 |
| g4201.t1 | MANBL | MANBAL | 0 | 16 |
| g9269.t1 | ZNT2 | Zinc transporter 2 | 2.30E-86 | 17 |
| g2221.t1 | S39AD | Zinc transporter ZIP13 | 2.18E-32 | 7 |
| g39450.t1 | S39AE | Zinc transporter ZIP14 | 3.64E-57 | 13 |
| g48890.t1 | BLM | Bloom syndrome homolog | 0 | 68 |
| g17088.t1 | SLAP2 | Src-like-adapter 2 | 1.82E-75 | 35 |
| g8802.t1 | LCLT1 | Lysocardiolipin acyltransferase 1 | 0 | 106 |
| g43919.t1 | S3TC1 | SH3 domain and tetratricopeptide repeat-containing 1 | 0 | 13 |
| g15928.t1 | S39AC | Zinc transporter ZIP12 | 6.65E-42 | 12 |
| g27825.t1 | S39A7 | Zinc transporter SLC39A7 | 5.86E-131 | 12 |
| g60221.t1 | S39A8 | Zinc transporter ZIP8 | 4.24E-98 | 17 |
| g51312.t1 | CUX1 | Homeobox cut-like 1 | 3.70E-31 | 6 |
| g16476.t1 | SOMA2 | Somatotropin-2 | 4.30E-54 | 6 |
| g14591.t1 | KCTD6 | BTB POZ domain-containing KCTD6 | 5.07E-136 | 12 |
| g54118.t1 | ITPR1 | Inositol 1,4,5-trisphosphate receptor type 1 | 0 | 52 |
| g36638.t1 | ITPR3 | Inositol 1,4,5-trisphosphate receptor type 3 | 7.79E-145 | 56 |
| g63653.t1 | ITPR2 | Inositol 1,4,5-trisphosphate receptor type 2 | 0 | 64 |
| g62059.t1 | TMCO3 | Transmembrane and coiled-coil domain-containing 3 | 1.82E-139 | 8 |
| g57304.t1 | KCY | UMP-CMP kinase | 0 | 11 |
| g14948.t1 | BCL3 | B-cell lymphoma 3 homolog | 4.22E-59 | 112 |
| g21203.t1 | BCL2 | Apoptosis regulator Bcl-2 | 2.13E-46 | 131 |
| g6919.t1 | PSPC1 | Paraspeckle component 1 | 0 | 60 |
| g902.t1 | WFS1 | Wolframin | 0 | 24 |
| g2368.t1 | WHAMM | WASP homolog-associated with membranes and microtubules | 0 | 30 |
| g11029.t1 | BAX | Apoptosis regulator BAX | 4.55E-18 | 69 |
| g63398.t1 | PTPS | 6-pyruvoyl tetrahydrobiopterin synthase | 3.36E-156 | 41 |
| g38601.t1 | PPA6 | Lysophosphatidic acid phosphatase type 6 Flags | 2.52E-131 | 24 |
| g1649.t1 | KLH17 | Kelch 17 | 0 | 28 |
| g39985.t1 | RHBL1 | Rhomboid-related 1 | 2.10E-146 | 8 |
| g54115.t1 | RHBG | Ammonium transporter Rh type B | 1.08E-142 | 8 |
| g11551.t1 | KLHL3 | Kelch 3 | 0 | 18 |
| g2490.t1 | KLHL4 | Kelch 4 | 0 | 14 |
| g29300.t1 | APOEB | Apolipo Eb | 6.88E-127 | 45 |
| g24007.t1 | S22AI | Solute carrier family 22 member 18 | 3.35E-121 | 11 |
| g56466.t1 | HDA10 | Histone deacetylase 10 | 0 | 75 |
| g30790.t1 | PICAL | Phosphatidylinositol-binding clathrin assembly | 0 | 50 |
| g24940.t1 | AP180 | Clathrin coat assembly AP180 | 0 | 50 |
| g46803.t1 | PICK1 | PRKCA-binding | 1.12E-122 | 53 |
| g8030.t1 | AP1AR | AP-1 complex-associated regulatory | 1.56E-23 | 53 |
| g55824.t1 | SG196 | O-mannose kinase | 0 | 6 |
| g33081.t1 | SFXN5 | Sideroflexin-5 | 2.32E-180 | 6 |
| g21292.t1 | TS101 | Tumor susceptibility gene 101 | 2.74E-48 | 12 |
| g24335.t1 | E2AK2 | Interferon- double-stranded RNA-activated kinase | 1.15E-81 | 55 |
| g3867.t1 | ANKAR | Ankyrin and armadillo repeat-containing | 0 | 80 |
| g898.t1 | ANK2 | Ankyrin-2 | 0 | 89 |
| g14256.t1 | ANK1 | Ankyrin-1 | 0 | 79 |
| g11931.t1 | NPAS3 | Neuronal PAS domain-containing 3 | 1.81E-36 | 31 |
| g15853.t1 | ANR50 | Ankyrin repeat domain-containing 50 | 1.36E-74 | 50 |
| g53450.t1 | L3BPA | Galectin-3-binding A | 4.08E-156 | 11 |
| g54813.t1 | DCC | Netrin receptor DCC | 7.13E-90 | 36 |
| g40708.t1 | GABP2 | GA-binding subunit beta-2 | 1.33E-135 | 29 |
| g15847.t1 | HPHL1 | Hephaestin 1 Flags | 0 | 12 |
| g3020.t1 | CFAI | Complement factor I | 8.77E-132 | 22 |
| g28365.t1 | HEMK1 | methyltransferase family member 1 | 0 | 26 |
| g53452.t1 | LAAT1 | Lysosomal amino acid transporter 1 homolog | 2.15E-122 | 12 |
| g34442.t1 | NEP | Neprilysin | 0 | 33 |
| g62824.t1 | SFXN2 | Sideroflexin-2 | 0 | 8 |
| g55966.t1 | SFXN1 | Sideroflexin-1 | 2.64E-32 | 8 |
| g24334.t1 | E2AK1 | Eukaryotic translation initiation factor 2-alpha kinase 1 | 1.93E-61 | 39 |
| g24780.t1 | OVOL1 | transcription factor Ovo-like 1 | 1.86E-178 | 7 |
| g30732.t1 | PRS35 | Inactive serine protease 35 Flags | 5.05E-27 | 26 |
| g28789.t1 | S39A4 | Zinc transporter ZIP4 | 1.08E-150 | 14 |
| g21014.t1 | ST17A | Serine threonine- kinase 17A | 0 | 14 |
| g1134.t1 | ST14 | Suppressor of tumorigenicity 14 homolog | 6.81E-154 | 12 |
| g23691.t1 | SREC | Scavenger receptor class F member 1 | 4.64E-25 | 26 |
| g33130.t1 | DMBTL | DMBT1 Flags | 1.61E-26 | 21 |
| g45606.t1 | SOGA1 | SOGA1 | 2.32E-137 | 46 |
| g59330.t1 | ERBB4 | Receptor tyrosine- kinase erbB-4 | 2.96E-37 | 125 |
| g56181.t1 | KCNA4 | Potassium voltage-gated channel subfamily A member 4 | 0 | 35 |
| g56180.t1 | KCNA5 | Potassium voltage-gated channel subfamily A member 5 | 0 | 32 |
| g16934.t1 | KCNA3 | Potassium voltage-gated channel subfamily A member 3 | 0 | 38 |
| g32682.t1 | KCNA2 | Potassium voltage-gated channel subfamily A member 2 | 1.01E-80 | 38 |
| g27623.t1 | KCNA1 | Potassium voltage-gated channel subfamily A member 1 | 0 | 38 |
| g34469.t1 | TRPM1 | Transient receptor potential cation channel subfamily M member 1 | 5.42E-114 | 28 |
| g16351.t1 | MTSS1 | Metastasis suppressor 1 | 0 | 31 |
| g25171.t1 | CNNM4 | Metal transporter CNNM4 | 3.14E-40 | 13 |
| g17860.t1 | MTU1 | Mitochondrial tRNA-specific 2-thiouridylase 1 | 1.20E-139 | 29 |
| g63828.t1 | TRPM2 | Transient receptor potential cation channel subfamily M member 2 | 0 | 30 |
| g26519.t1 | PSD12 | 26S proteasome non-ATPase regulatory subunit 12 | 1.52E-124 | 50 |
| g63228.t1 | AAA1 | Asc-type amino acid transporter 1 | 1.22E-63 | 20 |
| g5402.t1 | LAT3 | Large neutral amino acids transporter small subunit 3 | 0 | 24 |
| g276.t1 | LAT1 | Large neutral amino acids transporter small subunit 1 | 0 | 24 |
| g19077.t1 | YLPM1 | YLP motif-containing 1 | 0 | 19 |
| g53668.t1 | ANR28 | Serine threonine- phosphatase 6 regulatory ankyrin repeat subunit A | 0 | 36 |
| g46725.t1 | RIPL1 | RILP 1 | 1.77E-177 | 67 |
| g63230.t1 | AAAD | Arylacetamide deacetylase | 2.50E-174 | 25 |
| g47578.t1 | LAT2 | Large neutral amino acids transporter small subunit 2 | 0 | 26 |
| g28025.t1 | TNMD | Tenomodulin | 0 | 61 |
| g26627.t1 | MID51 | Mitochondrial dynamics MID51 | 4.16E-96 | 14 |
| g63605.t1 | AFP4 | Type-4 ice-structuring LS-12 | 0 | 27 |
| g18662.t1 | AFG32 | AFG3 2 | 0 | 27 |
| g53798.t1 | NCOA1 | Nuclear receptor coactivator 1 | 0 | 15 |
| g54537.t1 | NAC82 | NAC domain-containing 82 | 4.36E-125 | 34 |
| g60837.t1 | ANXA3 | Annexin A3 | 1.32E-32 | 40 |
| g64506.t1 | GNAT1 | Guanine nucleotide-binding G(t) subunit alpha-1 | 3.43E-51 | 34 |
| g21649.t1 | ASPX | Acrosomal SP-10 | 0 | 54 |
| g46936.t1 | ADRM1 | Proteasomal ubiquitin receptor ADRM1 | 7.33E-180 | 41 |
| g43205.t1 | EDN3 | Endothelin-3 | 1.71E-30 | 26 |
| g4187.t1 | ASPP1 | Apoptosis-stimulating of p53 1 | 0 | 36 |
| g48374.t1 | ASPP2 | Apoptosis-stimulating of p53 2 | 0 | 34 |
| g20917.t1 | IBP1 | Insulin-like growth factor-binding 1 | 1.74E-112 | 34 |
| g52348.t1 | HYAL3 | Hyaluronidase-3 | 3.81E-159 | 50 |
| g13066.t1 | HYAL2 | Hyaluronidase-2 | 1.57E-153 | 50 |
| g9120.t1 | BTBDA | BTB POZ domain-containing 10 | 0 | 18 |
| g55453.t1 | INF2 | Inverted formin-2 | 1.76E-49 | 23 |
| g5001.t1 | INE | Sodium- and chloride-dependent GABA transporter | 2.59E-134 | 52 |
| g59104.t1 | STK4 | Serine threonine- kinase 4 | 0 | 34 |
| g14764.t1 | OXSR1 | Serine threonine- kinase OSR1 | 0 | 33 |
| g41242.t1 | ADRB2 | Beta-2 adrenergic receptor | 0 | 47 |
| g31730.t1 | FGF3 | Fibroblast growth factor 3 | 1.75E-91 | 27 |
| g5126.t1 | GA2L1 | GAS2 1 | 0 | 11 |
| g10093.t1 | G6PT2 | Glucose-6-phosphate exchanger SLC37A1 | 2.98E-111 | 11 |
| g10095.t1 | G6PT3 | Glucose-6-phosphate exchanger SLC37A2 | 8.57E-107 | 11 |
| g51860.t1 | RNP1A | RNA-binding with serine-rich domain 1-A | 2.47E-159 | 12 |
| g63187.t1 | SPXN2 | Sperm associated with the nucleus on the X chromosome N2 | 0 | 5 |
| g34279.t1 | SFRP5 | Secreted frizzled-related 5 | 1.59E-156 | 46 |
| g27618.t1 | KCNB1 | Potassium voltage-gated channel subfamily B member 1 | 0 | 48 |
| g12298.t1 | KCNJ1 | ATP-sensitive inward rectifier potassium channel 1 | 1.53E-156 | 33 |
| g54563.t1 | KCJ16 | Inward rectifier potassium channel 16 | 5.77E-159 | 33 |
| g50913.t1 | KCJ11 | ATP-sensitive inward rectifier potassium channel 11 | 0 | 33 |
| g8515.t1 | KCJ10 | ATP-sensitive inward rectifier potassium channel 10 | 0 | 33 |
| g59081.t1 | KCNH7 | Potassium voltage-gated channel subfamily H member 7 | 0 | 23 |
| g2041.t1 | KCNH6 | Potassium voltage-gated channel subfamily H member 6 | 0 | 25 |
| g23988.t1 | KCNH4 | Potassium voltage-gated channel subfamily H member 4 | 0 | 25 |
| g14781.t1 | KCNH2 | Potassium voltage-gated channel subfamily H member 2 | 0 | 25 |
| g41314.t1 | MAG | Myelin-associated glyco | 2.00E-53 | 18 |
| g37490.t1 | MAFK | Transcription factor | 2.00E-53 | 18 |
| g10887.t1 | MAFG | Transcription factor | 5.44E-100 | 16 |
| g55829.t1 | S4A11 | Sodium bicarbonate transporter 11 | 0 | 20 |
| g12933.t1 | ACADS | -chain specific acyl- mitochondrial | 0 | 28 |
| g2495.t1 | SELTA | Seleno T1a | 6.75E-75 | 30 |
| g46700.t1 | FXA4B | Forkhead box A4-B | 2.62E-102 | 23 |
| g26659.t1 | FOXO3 | Forkhead box O3 | 2.53E-113 | 29 |
| g63497.t1 | S35C2 | Solute carrier family 35 member C2 | 0 | 21 |
| g55918.t1 | DGKZ | Diacylglycerol kinase zeta | 0 | 42 |
| g61655.t1 | DAZP2 | DAZ-associated 2 | 4.44E-162 | 26 |
| g32841.t1 | MPCP | Phosphate carrier mitochondrial | 4.62E-67 | 9 |
| g24834.t1 | CADH1 | Cadherin-1 | 0 | 62 |
| g23572.t1 | CADM1 | Cell adhesion molecule 1 | 3.99E-63 | 77 |
| g28436.t1 | ACON | Aconitate mitochondrial | 0 | 17 |
| g61248.t1 | SCEL | Sciellin | 0 | 17 |
| g36726.t1 | SCD5 | Stearoyl- desaturase 5 | 0 | 17 |
| g52761.t1 | GLCNE | Bifunctional UDP-N-acetylglucosamine 2-epimerase N-acetylmannosamine kinase | 0 | 32 |
| g23283.t1 | GLCM | Glucosylceramidase | 0 | 32 |
| g3975.t1 | SRF | Serum response factor | 2.05E-94 | 97 |
| g48363.t1 | SRFB1 | Serum response factor-binding 1 | 6.46E-84 | 92 |
| g198.t1 | EFCB6 | EF-hand calcium-binding domain-containing 6 | 7.07E-80 | 12 |
| g42090.t1 | ADCY8 | Adenylate cyclase type 8 | 5.54E-174 | 35 |
| g48174.t1 | ADCY2 | Adenylate cyclase type 2 | 0 | 24 |
| g61855.t1 | ADCY3 | Adenylate cyclase type 3 | 0 | 23 |
| g34395.t1 | MYO5A | Unconventional myosin-Va | 0 | 71 |
| g20581.t1 | MYO5C | Unconventional myosin-Vc | 5.06E-141 | 86 |
| g62188.t1 | MYO5B | Unconventional myosin-Vb | 0 | 75 |
| g54211.t1 | V2R1 | Vomeronasal type-2 receptor 1 | 4.12E-51 | 49 |
| g32553.t1 | CP4V2 | Cytochrome P450 4V2 | 0 | 32 |
| g38188.t1 | KASH5 | KASH5 | 0 | 65 |
| g59661.t1 | KAPCB | cAMP-dependent kinase catalytic subunit beta | 0 | 65 |
| g16732.t1 | KAPCA | cAMP-dependent kinase catalytic subunit alpha | 0 | 65 |
| g58047.t1 | PRL | Prolactin | 1.24E-124 | 50 |
| g38898.t1 | ADCYA | Adenylate cyclase type 10 | 0 | 22 |
| g38894.t1 | ADCY9 | Adenylate cyclase type 9 | 0 | 18 |
| g49308.t1 | ADCY5 | Adenylate cyclase type 5 | 0 | 34 |
| g36835.t1 | MYO10 | Unconventional myosin-X | 3.88E-79 | 39 |
| g7483.t1 | V2R | Vasopressin V2 receptor | 9.02E-81 | 30 |
| g63178.t1 | AK1D1 | 3-oxo-5-beta-steroid 4-dehydrogenase | 1.84E-158 | 55 |
| g63177.t1 | ALG1 | Chitobiosyldiphosphodolichol beta-mannosyl transferase | 1.93E-156 | 47 |
| g30123.t1 | ADCY1 | Adenylate cyclase type 1 | 0 | 39 |
| g7465.t1 | ADCY6 | Adenylate cyclase type 6 | 0 | 37 |
| g1589.t1 | CLCN1 | Chloride channel 1 | 1.53E-43 | 13 |
| g6954.t1 | H48 | Histone H4 type VIII | 1.66E-22 | 33 |
| g38004.t1 | GIMA8 | GTPase IMAP family member 8 | 1.39E-48 | 18 |
| g45657.t1 | LRP12 | Low-density lipo receptor-related 12 | 8.52E-100 | 16 |
| g31291.t1 | CREG1 | CREG1 Flags | 2.44E-65 | 36 |
| g7842.t1 | NFYB | Nuclear transcription factor Y subunit beta | 6.71E-137 | 22 |
| g17988.t1 | ULK1 | Serine threonine- kinase ULK1 | 0 | 19 |
| g18213.t1 | FNTB | farnesyltransferase subunit beta | 1.89E-168 | 38 |
| g6757.t1 | GIMA4 | GTPase IMAP family member 4 | 5.30E-24 | 17 |
| g3595.t1 | TICN1 | Testican-1 | 4.38E-102 | 33 |
| g28040.t1 | RPKL1 | Ribosomal S6 kinase-like 1 | 1.05E-18 | 37 |
| g28044.t1 | YAED1 | Yae1 domain-containing 1 | 6.83E-17 | 32 |
| g64211.t1 | H5 | Histone H5 | 6.00E-56 | 48 |
| g64309.t1 | H4 | Histone H4 | 2.03E-67 | 48 |
| g832.t1 | CD9 | CD9 antigen | 1.78E-104 | 24 |
| g33350.t1 | FOCAD | Focadhesin | 0 | 22 |
| g3274.t1 | TYB11 | Thymosin beta-11 | 1.01E-144 | 85 |
| g25006.t1 | CATQ | Cathepsin Q Flags | 1.51E-06 | 36 |
| g12552.t1 | MARH7 | E3 ubiquitin- ligase MARCH7 | 6.53E-26 | 17 |
| g38507.t1 | PE2R4 | Prostaglandin E2 receptor EP4 subtype | 1.53E-173 | 61 |
| g19323.t1 | PSN2 | Presenilin-2 | 0 | 92 |
| g61730.t1 | P2RX4 | P2X purinoceptor 4 | 1.06E-25 | 63 |
| g43164.t1 | CTGF | Connective tissue growth factor | 1.16E-143 | 69 |
| g40036.t1 | CYR61 | CYR61 | 4.39E-62 | 52 |
| g33563.t1 | WISP3 | WNT1-inducible-signaling pathway 3 | 3.69E-142 | 34 |
| g30866.t1 | WISP1 | WNT1-inducible-signaling pathway 1 | 3.90E-144 | 34 |
| g45660.t1 | CTHR1 | Collagen triple helix repeat-containing 1 Flags | 0 | 72 |
| g33598.t1 | NOVA1 | RNA-binding Nova-1 | 1.22E-133 | 62 |
| g38559.t1 | RSU1 | Ras suppressor 1 | 6.80E-120 | 35 |
| g25698.t1 | RB11A | Ras-related Rab-11A Flags | 1.42E-174 | 49 |
| g25693.t1 | RB | Retinoblastoma-associated | 7.74E-144 | 52 |
| g12774.t1 | ANR16 | Ankyrin repeat domain-containing 16 | 0 | 32 |
| g24561.t1 | STK11 | Serine threonine- kinase STK11 | 7.34E-172 | 56 |
| g12707.t1 | CO2A1 | Collagen alpha-1(II) chain | 1.04E-172 | 42 |
| g48736.t1 | WIZ | Wiz | 1.60E-146 | 28 |
| g50769.t1 | FGF20 | Fibroblast growth factor 20 | 7.89E-80 | 78 |
| g11609.t1 | SLF2 | SMC5-SMC6 complex localization factor 2 | 1.33E-52 | 43 |
| g20360.t1 | ANR39 | Ankyrin repeat domain-containing 39 | 2.56E-44 | 32 |
| g18587.t1 | ANR12 | Ankyrin repeat domain-containing 12 | 0 | 27 |
| g8982.t1 | TOP1 | DNA topoisomerase 1 | 0 | 24 |
| g48058.t1 | BCOR | BCL-6 corepressor | 1.70E-137 | 39 |
| g32986.t1 | BCR | Breakpoint cluster region | 1.02E-110 | 28 |
| g26805.t1 | EZRI | Ezrin | 0 | 41 |
| g53238.t1 | EZH2 | Histone-lysine N-methyltransferase EZH2 | 0 | 42 |
| g64315.t1 | CYSP1 | Digestive cysteine ase 1 Flags | 6.40E-108 | 46 |
| g16522.t1 | CO1A2 | Collagen alpha-2(I) chain | 1.38E-160 | 68 |
| g12524.t1 | CO1A1 | Collagen alpha-1(I) chain | 1.60E-143 | 68 |
| g54102.t1 | CO3 | Complement C3 Contains | 1.27E-29 | 77 |
| g13742.t1 | CO6 | Complement component C6 Flags | 5.47E-95 | 35 |
| g36376.t1 | HXD1 | Homeobox Hox-D1 | 7.19E-51 | 26 |
| g31281.t1 | HXB1 | Homeobox Hox-B1 | 1.13E-161 | 23 |
| g39839.t1 | SCYL2 | SCY1 2 | 4.45E-65 | 53 |
| g27417.t1 | TCF15 | Transcription factor 15 | 4.87E-32 | 60 |
| g41939.t1 | TCF23 | Transcription factor 23 | 4.75E-66 | 55 |
| g54884.t1 | TCF19 | Transcription factor 19 | 1.19E-78 | 63 |
| g30887.t1 | SCX | Basic helix-loop-helix transcription factor scleraxis | 1.36E-84 | 91 |
| g44478.t1 | PGFRL | Platelet-derived growth factor receptor | 0 | 80 |
| g5321.t1 | PGFRB | Platelet-derived growth factor receptor beta | 0 | 81 |
| g39086.t1 | TRI32 | E3 ubiquitin- ligase TRIM32 | 0 | 40 |
| g26428.t1 | P2RX3 | P2X purinoceptor 3 | 3.56E-35 | 58 |
| g4711.t1 | ACAC | Acetyl- carboxylase | 0 | 34 |
| g57243.t1 | ACAD8 | Isobutyryl- mitochondrial | 0 | 35 |
| g12355.t1 | ACACB | Acetyl- carboxylase 2 | 0 | 35 |
| g51190.t1 | OLA1 | Obg-like ATPase 1 | 0 | 10 |
| g52500.t1 | SCM2B | Calcium-binding mitochondrial carrier S -2-B | 0 | 20 |
| g30020.t1 | SCMC3 | Calcium-binding mitochondrial carrier S -3 | 0 | 17 |
| g53272.t1 | SCMH1 | Polycomb SCMH1 | 0 | 16 |
| g47139.t1 | FUBP2 | Far upstream element-binding 2 | 0 | 29 |
| g30085.t1 | GHRL | Ghrelin Contains | 2.60E-116 | 29 |
| g21067.t1 | UT14A | U3 small nucleolar RNA-associated 14 homolog A | 3.80E-102 | 7 |
| g57035.t1 | UTP11 | Probable U3 small nucleolar RNA-associated 11 | 1.42E-158 | 8 |
| g31925.t1 | MYNN | Myoneurin | 6.65E-147 | 35 |
| g36458.t1 | MYLK3 | Myosin light chain kinase 3 | 6.26E-174 | 34 |
| g16456.t1 | MIP18 | Mitotic spindle-associated MMXD complex subunit MIP18 | 2.85E-32 | 21 |
| g26942.t1 | NHS | Nance-Horan syndrome | 3.56E-13 | 22 |
| g30446.t1 | PRKRA | Interferon-inducible double-stranded RNA-dependent kinase activator A homolog | 0 | 111 |
| g30445.t1 | PRKN | E3 ubiquitin- ligase parkin | 8.52E-16 | 112 |

**Table S18 . Total 63 genes in cellular calcium ion homeostasis GO:0006874 in Hilsa**

| **S No** | **Gene ID** | **No of copies** | **Gene description** |
| --- | --- | --- | --- |
|  | KPCB | 9 | kinase C beta type |
|  | NCKX3 | 6 | Sodium potassium calcium exchanger 3 |
|  | GRIK2 | 6 | Glutamate receptor kainate 2 |
|  | ACHA7 | 4 | Neuronal acetylcholine receptor subunit alpha-7 Flags: Precursor |
|  | GPR98 | 4 | G- coupled receptor 98 |
|  | AT133 | 3 | Probable cation-transporting ATPase 13A3 |
|  | CSTN2 | 3 | Calsyntenin-2 |
|  | GLRK | 3 | Probable glutamate receptor |
|  | GLRX | 3 | Glutaredoxin |
|  | GPC6A | 3 | G- coupled receptor family C group 6 member A |
|  | KPCA | 3 | kinase C alpha type |
|  | NCKX2 | 3 | Sodium potassium calcium exchanger 2 |
|  | CCL4 | 2 | C-C motif chemokine 4 homolog |
|  | GRIK1 | 2 | Glutamate receptor kainate 1 |
|  | GRIK4 | 2 | Glutamate receptor kainate 4 |
|  | MRCKA | 2 | Serine threonine- kinase MRCK alpha |
|  | NCKX4 | 2 | Sodium potassium calcium exchanger 4 |
|  | NMDZ1 | 2 | Glutamate receptor NMDA 1 |
|  | STIM2 | 2 | Stromal interaction molecule 2 Flags: Precursor |
|  | SV2B | 2 | Synaptic vesicle glyco 2B |
|  | ACHA9 | 1 | Neuronal acetylcholine receptor subunit alpha-9 |
|  | ANX13 | 1 | Annexin A13 Intestine-specific annexin |
|  | AT132 | 1 | Cation-transporting ATPase 13A2 |
|  | AT1B2 | 1 | Sodium potassium-transporting ATPase subunit beta-2 |
|  | AT2A1 | 1 | Sarcoplasmic endoplasmic reticulum calcium ATPase 1 |
|  | B2CL1 | 1 | Bcl-2 1 |
|  | CASR | 1 | Extracellular calcium-sensing receptor |
|  | CCL11 | 1 | Eotaxin |
|  | CRFR1 | 1 | Corticotropin-releasing factor receptor 1 |
|  | GCN1 | 1 | eIF-2-alpha kinase activator GCN1 |
|  | GRIK5 | 1 | Glutamate receptor kainate 5 |
|  | HEXA | 1 | Beta-hexosaminidase subunit alpha |
|  | HEXB | 1 | Beta-hexosaminidase subunit beta |
|  | HEXDC | 1 | Hexosaminidase D |
|  | ITA3 | 1 | Integrin alpha-3 |
|  | ITA6 | 1 | Integrin alpha-6 |
|  | KPCD | 1 | kinase C delta type |
|  | NALCN | 1 | Sodium leak channel non-selective |
|  | NCKX5 | 1 | Sodium potassium calcium exchanger 5 |
|  | NMI | 1 | N-myc-interactor |
|  | PK3CA | 1 | Phosphatidylinositol 4,5-bisphosphate 3-kinase catalytic subunit alpha isoform |
|  | PK3CB | 1 | Phosphatidylinositol 4,5-bisphosphate 3-kinase catalytic subunit beta isoform |
|  | PK3CD | 1 | Phosphatidylinositol 4,5-bisphosphate 3-kinase catalytic subunit delta isoform |
|  | PK3CG | 1 | Phosphatidylinositol 4,5-bisphosphate 3-kinase catalytic subunit gamma isoform |
|  | PTHB1 | 1 | PTHB1 |
|  | PTHY | 1 | Parathyroid hormone |
|  | PTK6 | 1 | -tyrosine kinase 6 |
|  | PYGL | 1 | Glycogen liver form |
|  | PYGM | 1 | Glycogen muscle form |
|  | PYGO1 | 1 | Pygopus homolog 1 |
|  | SSR1 | 1 | Somatostatin receptor type 1 |
|  | STC | 1 | Stanniocalcin |
|  | STC2 | 1 | Stanniocalcin-2 |
|  | STIM1 | 1 | Stromal interaction molecule 1 Flags: Precursor |
|  | STMN1 | 1 | Stathmin |
|  | SYPM | 1 | Probable proline--tRNA mitochondrial |
|  | TGFB2 | 1 | Transforming growth factor beta-2 |
|  | TGM5 | 1 | -glutamine gamma-glutamyltransferase 5 |
|  | TNR5 | 1 | Tumor necrosis factor receptor superfamily member 5 |
|  | VEGFC | 1 | Vascular endothelial growth factor C |
|  | WWP1 | 1 | NEDD4-like E3 ubiquitin- ligase WWP1 |
|  | ZNT1 | 1 | Zinc transporter 1 |
|  | ZNT10 | 1 | Zinc transporter 10 |

**Table S19 . Total 51 genes in tissue homeostasis** **GO:0001894 pertaining to osmo-regulation in Hilsa**

| S No | Gene ID | No of copies | Gene description | e-value |
| --- | --- | --- | --- | --- |
|  | ABCB6 | 1 | ATP-binding cassette sub-family B member mitochondrial | 0 |
|  | ACAC | 1 | Acetyl- carboxylase | 0 |
|  | ACACB | 1 | Acetyl- carboxylase 2 | 0 |
|  | ACAD8 | 1 | Isobutyryl- mitochondrial | 0 |
|  | AK1D1 | 1 | 3-oxo-5-beta-steroid 4-dehydrogenase | 1.84E-158 |
|  | ALG1 | 1 | Chitobiosyldiphosphodolichol beta-mannosyltransferase | 1.93E-156 |
|  | ANR12 | 1 | Ankyrin repeat domain-containing 12 | 0 |
|  | ANR16 | 1 | Ankyrin repeat domain-containing 16 | 0 |
|  | ANR39 | 1 | Ankyrin repeat domain-containing 39 | 2.56E-44 |
|  | BCOR | 1 | BCL-6 corepressor | 1.70E-137 |
|  | BCR | 1 | Breakpoint cluster region | 1.02E-110 |
|  | CO1A1 | 3 | Collagen alpha-1(I) chain | 1.60E-143 |
|  | CO1A2 | 1 | Collagen alpha-2(I) chain | 1.38E-160 |
|  | CO2A1 | 5 | Collagen alpha-1(II) chain | 1.04E-172 |
|  | CO3 | 1 | Complement C3 Contains | 1.27E-29 |
|  | CO6 | 1 | Complement component C6 Flags: Precursor | 5.47E-95 |
|  | CTGF | 1 | Insulin-like growth factor-binding 8 | 1.16E-143 |
|  | CTHR1 | 1 | Collagen triple helix repeat-containing 1 Flags: Precursor | 0 |
|  | CYR61 | 6 | Cysteine-rich angiogenic inducer 61 Flags: Precursor | 3.92E-142 |
|  | CYSP1 | 1 | Digestive cysteine ase 1 Flags: Precursor | 6.40E-108 |
|  | EZH2 | 1 | Histone-lysine N-methyltransferase Enhancer of zeste homolog 2 | 0 |
|  | EZRI | 1 | Ezrin Cytovillin | 0 |
|  | FGF20 | 1 | Fibroblast growth factor 20 | 7.89E-80 |
|  | GNAS | 4 | Adenylate cyclase-stimulating Guanine nucleotide-binding G(s) subunit alpha | 4.71E-98 |
|  | HXB1 | 1 | Homeobox Hox-B1 | 1.13E-161 |
|  | HXD1 | 1 | Homeobox Hox-D1 | 7.19E-51 |
|  | KCJ10 | 1 | ATP-sensitive inward rectifier potassium channel 10 | 0 |
|  | KCJ11 | 1 | ATP-sensitive inward rectifier potassium channel 11 | 0 |
|  | KCJ16 | 1 | Inward rectifier potassium channel 16 | 5.77E-159 |
|  | KCNJ1 | 1 | ATP-sensitive inward rectifier potassium channel 1 | 1.53E-156 |
|  | KCNJ2 | 1 | Inward rectifier potassium channel 2 | 1.93E-159 |
|  | NOVA1 | 1 | RNA-binding Neuro-oncological ventral antigen 1 | 1.22E-133 |
|  | P2RX3 | 1 | P2X purinoceptor 3 | 3.56E-35 |
|  | P2RX4 | 2 | P2X purinoceptor 4 | 0 |
|  | PGFRB | 1 | Platelet-derived growth factor receptor beta | 0 |
|  | PGFRL | 1 | Platelet-derived growth factor receptor | 0 |
|  | RB | 1 | Retinoblastoma-associated | 7.74E-144 |
|  | RB11A | 1 | Ras-related Rab-11A Flags: Precursor | 1.42E-174 |
|  | RSU1 | 1 | Ras suppressor 1 | 6.80E-120 |
|  | SCX | 1 | Basic helix-loop-helix transcription factor scleraxis | 1.36E-84 |
|  | SCYL2 | 1 | Coated vesicle-associated kinase of 104 kDa | 4.45E-65 |
|  | SLF2 | 1 | SMC5-SMC6 complex localization factor 2 | 1.33E-52 |
|  | STK11 | 2 | Serine threonine- kinase STK11 | 7.34E-172 |
|  | TCF15 | 1 | Transcription factor 15 | 4.87E-32 |
|  | TCF19 | 1 | Transcription factor 19 | 1.19E-78 |
|  | TCF23 | 1 | Transcription factor 23 | 4.75E-66 |
|  | TOP1 | 1 | DNA topoisomerase 1 | 0 |
|  | TRI32 | 1 | E3 ubiquitin- ligase TRIM32 | 0 |
|  | WISP1 | 1 | WNT1-inducible-signaling pathway 4 | 3.90E-144 |
|  | WISP3 | 1 | WNT1-inducible-signaling pathway 3 | 3.69E-142 |
|  | WIZ | 1 | Widely-interspaced zinc finger-containing | 1.60E-146 |

**Table S20 . Total 48 genes pertaining to cellular iron ion homeostasis GO 0006879 in Hilsa**

| **S. No.** | **Description** |  | **Gene Description** | **e-Value** |
| --- | --- | --- | --- | --- |
|  | ABCB6 | 2 | ATP-binding cassette sub-family B member mitochondrial | 2.95E-73 |
|  | ABCB7 | 1 | ATP-binding cassette sub-family B member mitochondrial | 0 |
|  | ABCB8 | 1 | ATP-binding cassette sub-family B member mitochondrial Flags: Precursor | 0 |
|  | ABCG2 | 3 | ATP-binding cassette sub-family G member 2 | 0 |
|  | ABCG4 | 1 | ATP-binding cassette sub-family G member 4 | 2.24E-79 |
|  | ACOC | 1 | Cytoplasmic aconitate hydratase | 0 |
|  | ACOD | 1 | Acyl- desaturase | 4.59E-70 |
|  | ADIPO | 1 | Adiponectin | 7.74E-15 |
|  | ALRF2 | 1 | Aly REF export factor 2 | 1.30E-70 |
|  | AT132 | 1 | Cation-transporting ATPase 13A2 | 0 |
|  | AT133 | 2 | Probable cation-transporting ATPase 13A3 | 0 |
|  | ATP23 | 1 | Mitochondrial inner membrane protease ATP23 homolog | 5.51E-29 |
|  | CERU | 2 | Ferroxidase Flags: Precursor, Ceruloplasmin | 3.13E-172 |
|  | CETA | 1 | 2-epi-5-epi-valiolone synthase | 0 |
|  | EGLN1 | 2 | Hypoxia-inducible factor prolyl hydroxylase 2, Egl nine homolog 1 | 2.37E-80 |
|  | EGLN3 | 1 | Hypoxia-inducible factor prolyl hydroxylase 3, Egl nine homolog 3 | 7.73E-79 |
|  | EGR1 | 1 | Early growth response 1 | 2.30E-118 |
|  | EPB1B | 1 | Ephrin type-B receptor 1-B | 0 |
|  | FMC1 | 1 | FMC1 homolog | 0 |
|  | FREM1 | 1 | FRAS1-related extracellular matrix 1 | 1.88E-57 |
|  | FRIH | 1 | Ferritin H heavy subunit | 1.62E-69 |
|  | HEM0 | 1 | 5-aminolevulinate erythroid- mitochondrial | 7.43E-68 |
|  | HEM1 | 1 | 5-aminolevulinate mitochondrial | 0 |
|  | HEPS | 1 | Serine protease hepsin Contains: | 1.80E-22 |
|  | HIF1A | 3 | Hypoxia-inducible factor 1-alpha | 4.34E-148 |
|  | HMOX | 1 | Heme oxygenase | 1.49E-110 |
|  | HMOX2 | 1 | Heme oxygenase 2 | 3.06E-119 |
|  | HMR1 | 1 | Major histocompatibility complex class I-related gene | 3.53E-131 |
|  | HPLN1 | 1 | Hyaluronan and proteoglycan link 1 | 0 |
|  | IRF1 | 1 | Interferon regulatory factor 1 | 0 |
|  | ISK1 | 1 | Probable pancreatic secretory ase inhibitor | 1.35E-84 |
|  | NADL2 | 1 | Inactive N-acetylated-alpha-linked acidic dipeptidase 2 | 0 |
|  | NRAP | 1 | Nebulin-related-anchoring | 0 |
|  | PCFT | 1 | Proton-coupled folate transporter , Heme carrier 1 | 0 |
|  | PRSS8 | 2 | Channel-activating protease 1, Prostasin | 1.60E-25 |
|  | S39A1 | 1 | Zinc transporter ZIP1, Solute carrier family 39 member 1 | 2.05E-93 |
|  | S39A3 | 1 | Zinc transporter ZIP3, Solute carrier family 39 member 3 | 5.36E-131 |
|  | S40A1 | 3 | Solute carrier family 40 member 1 | 0 |
|  | S41A1 | 3 | Solute carrier family 41 member 1 | 0 |
|  | SCAR5 | 1 | Scavenger receptor class A member 5 | 0 |
|  | SIM2 | 1 | Single-minded homolog 2 | 0 |
|  | SIM24 | 1 | Small integral membrane 24 Flags: Precursor | 9.28E-136 |
|  | SMAD4 | 4 | MAD Mothers against decapentaplegic homolog 4 | 8.76E-34 |
|  | SMAD5 | 1 | MAD Mothers against decapentaplegic homolog 5 | 0 |
|  | SODE | 1 | Extracellular superoxide dismutase | 2.46E-90 |
|  | TMPS6 | 3 | Transmembrane protease serine 6, Matriptase-2 | 2.22E-36 |
|  | TMPS7 | 1 | Transmembrane protease serine 7, Matriptase-3 | 3.77E-34 |
|  | TMPS9 | 2 | Transmembrane protease serine, Polyserase-I | 8.84E-49 |

**Table S21a. GOs and number of genes** **for water homeostasis in Hilsa**

| **S No** | **GO_ID** | **GO_Description** | **No of Genes** |
| --- | --- | --- | --- |
| **1** | **GO:0003091** | renal water homeostasis | 27 |
| **2** | **GO:0009414** | response to water deprivation | 25 |
| **3** | **GO:0009992** | cellular water homeostasis | 15 |
| **4** | **GO:0070295** | renal water absorption | 11 |
| **5** | **GO:0033561** | regulation of water loss via skin | 10 |
| **6** | **GO:0015250** | water channel activity | 10 |
| **7** | **GO:0006833** | water transport | 9 |
| **8** | **GO:0050891** | multicellular organismal water homeostasis | 8 |
| **9** | **GO:0042631** | cellular response to water deprivation | 4 |
| **10** | **GO:0003097** | renal water transport | 3 |
| **11** | **GO:0035377** | Trans-epithelial water transport | 3 |
| **12** | **GO:0030104** | water homeostasis | 3 |
| **13** | **GO:0005372** | water trans-membrane transporter activity | 3 |
| **14** | **GO:2000070** | regulation of response to water deprivation | 2 |
| **15** | **GO:0050824** | obsolete water binding | 1 |
| **16** | **GO:1990785** | response to water-immersion restraint stress | 1 |

**Table S21b. Total 97 Genes pertaining to water homeostasis** (refer table s21a) **in Hilsa**

|  | **Length (bp)** | **Gene_ID** | **Gene description** | **No. of copies** | **Organism** | **e-Value** |
| --- | --- | --- | --- | --- | --- | --- |
|  | 463 | ABCG4 | ATP-binding cassette sub-family G member 4 | 1 | HUMAN | 2.24E-79 |
|  | 328 | ACOD | Acyl- desaturase | 1 | CYPCA | 0 |
|  | 348 | ACON | Aconitate mitochondrial | 1 | BOVIN | 0 |
|  | 1098 | ADCY1 | Adenylate cyclase type 1 | 1 | MOUSE | 0 |
|  | 1303 | ADCY2 | Adenylate cyclase type 2 | 4 | RAT | 0 |
|  | 1040 | ADCY3 | Adenylate cyclase type 3 | 2 | RAT | 0 |
|  | 858 | ADCY5 | Adenylate cyclase type 5 | 2 | HUMAN | 0 |
|  | 1072 | ADCY6 | Adenylate cyclase type 6 | 1 | HUMAN | 0 |
|  | 488 | ADCY8 | Adenylate cyclase type 8 | 9 | HUMAN | 5.54E-174 |
|  | 907 | ADCY9 | Adenylate cyclase type 9 | 2 | CHICK | 0 |
|  | 648 | ADCYA | Adenylate cyclase type 10 | 1 | RABIT | 0 |
|  | 396 | ADRB2 | Beta-2 adrenergic receptor | 1 | ONCMY | 0 |
|  | 858 | AK1D1 | 3-oxo-5-beta-steroid 4-dehydrogenase | 1 | HUMAN | 1.84E-158 |
|  | 316 | ALG1 | Chitobiosyldiphosphodolichol beta-mannosyltransferase | 1 | HUMAN | 1.93E-156 |
|  | 316 | ANX13 | Annexin A13 | 1 | HUMAN | 8.57E-117 |
|  | 424 | AP2A1 | AP-2 complex subunit alpha-1 | 1 | HUMAN | 0 |
|  | 459 | AP2B1 | AP-2 complex subunit beta | 1 | RAT | 0 |
|  | 258 | AQP1 | Aquaporin-1 | 4 | RAT | 4.66E-115 |
|  | 94 | AQP10 | Aquaporin-10 | 1 | HUMAN | 7.19E-27 |
|  | 195 | AQP11 | Aquaporin-11 | 2 | HUMAN | 7.41E-52 |
|  | 304 | AQP3 | Aquaporin-3 | 1 | RAT | 2.49E-144 |
|  | 295 | AQP4 | Aquaporin-4 | 3 | BOVIN | 5.61E-106 |
|  | 212 | AQP7 | Aquaporin-7 | 1 | MOUSE | 6.14E-63 |
|  | 548 | AQP8 | Aquaporin-8 | 3 | RAT | 5.53E-95 |
|  | 116 | AQP9 | Aquaporin-9 | 3 | HUMAN | 9.24E-41 |
|  | 289 | AQR | Intron-binding aquarius | 1 | HUMAN | 5.90E-118 |
|  | 883 | CADH1 | Cadherin-1 | 4 | CANFA | 0 |
|  | 388 | CADM1 | Cell adhesion molecule 1 | 1 | HUMAN | 3.99E-63 |
|  | 121 | CATQ | Cathepsin Q Flags: Precursor | 1 | RAT | 1.51E-06 |
|  | 227 | CD9 | CD9 antigen ame: CD | 1 | RAT | 1.78E-104 |
|  | 187 | CLCN1 | Chloride channel 1 | 1 | MOUSE | 1.53E-43 |
|  | 558 | CP4V2 | Cytochrome P450 4V2 | 1 | HUMAN | 0 |
|  | 575 | CREG1 | CREG1 Flags: Precursor | 1 | CHICK | 2.44E-65 |
|  | 453 | DAZP2 | DAZ-associated 2 | 1 | HUMAN | 4.44E-162 |
|  | 1072 | EFCB6 | EF-hand calcium-binding domain-containing 6 | 1 | MACFA | 7.07E-80 |
|  | 375 | FNTB | farnesyltransferase subunit beta | 2 | MOUSE | 1.89E-168 |
|  | 429 | FOCAD | Focadhesin | 1 | HUMAN | 0 |
|  | 366 | GHRL | Ghrelin Contains: | 1 | ONCMY | 2.60E-116 |
|  | 639 | GIMA4 | GTPase IMAP family member 4 | 1 | MOUSE | 5.30E-24 |
|  | 1616 | GIMA8 | GTPase IMAP family member 8 | 3 | HUMAN | 1.39E-48 |
|  | 516 | GLCM | Glucosylceramidase | 1 | PONAB | 0 |
|  | 564 | GLCNE | Bifunctional UDP-N-acetylglucosamine 2-epimerase N-acetylmannosamine kinase | 1 | HUMAN | 0 |
|  | 140 | GNAS | Guanine nucleotide-binding G(s) subunit alpha | 4 | XENLA | 4.71E-98 |
|  | 103 | H4 | Histone H4 | 30 | SOLLC | 2.03E-67 |
|  | 81 | H48 | Histone H4 type VIII | 1 | CHICK | 1.66E-22 |
|  | 108 | H5 | Histone H5 | 1 | CHICK | 6.00E-56 |
|  | 492 | HYAL2 | Hyaluronidase-2 | 3 | HUMAN | 7.56E-128 |
|  | 531 | HYAL3 | Hyaluronidase-3 | 1 | MOUSE | 3.81E-159 |
|  | 468 | INE | Sodium- and chloride-dependent GABA transporter ine | 1 | DROME | 2.59E-134 |
|  | 762 | INF2 | Inverted formin-2 | 1 | HUMAN | 1.76E-49 |
|  | 713 | KAPCA | cAMP-dependent kinase catalytic subunit alpha | 1 | PIG | 0 |
|  | 351 | KAPCB | cAMP-dependent kinase catalytic subunit beta | 2 | HUMAN | 0 |
|  | 351 | KASH5 | KASH5 | 1 | BOVIN | 0 |
|  | 537 | LRP12 | Low-density lipo receptor-related 12 | 1 | PONAB | 8.52E-100 |
|  | 171 | MARH7 | E3 ubiquitin- ligase MARCH7 | 1 | PONAB | 6.53E-26 |
|  | 263 | MIP | Lens fiber major intrinsic | 3 | LITPI | 5.45E-149 |
|  | 264 | MIP18 | Mitotic spindle-associated MMXD complex subunit MIP18 | 1 | HUMAN | 2.85E-32 |
|  | 123 | MPCP | Phosphate carrier mitochondrial | 1 | MOUSE | 4.62E-67 |
|  | 607 | MYLK3 | Myosin light chain kinase 3 | 1 | DANRE | 6.26E-174 |
|  | 839 | MYNN | Myoneurin | 1 | DANRE | 6.65E-147 |
|  | 930 | MYO10 | Unconventional myosin-X | 1 | MOUSE | 3.88E-79 |
|  | 1996 | MYO5A | Unconventional myosin-Va | 2 | RAT | 0 |
|  | 1654 | MYO5B | Unconventional myosin-Vb | 1 | MOUSE | 0 |
|  | 282 | MYO5C | Unconventional myosin-Vc | 2 | HUMAN | 5.06E-141 |
|  | 1148 | NEDD1 | NEDD1 | 1 | HUMAN | 0 |
|  | 1295 | NEDD4 | E3 ubiquitin- ligase NEDD4 | 1 | MOUSE | 0 |
|  | 350 | NFYB | Nuclear transcription factor Y subunit beta | 1 | RAT | 6.71E-137 |
|  | 90 | NHS | Nance-Horan syndrome | 1 | HUMAN | 3.56E-13 |
|  | 533 | OXSR1 | Serine threonine- kinase OSR1 | 1 | PIG | 0 |
|  | 545 | P | P | 1 | MOUSE | 0 |
|  | 379 | PE2R4 | Prostaglandin E2 receptor EP4 subtype | 1 | RABIT | 1.53E-173 |
|  | 228 | PRL | Prolactin | 1 | CORAU | 1.24E-124 |
|  | 1556 | RECQ5 | ATP-dependent DNA helicase Q5 | 2 | HUMAN | 1.86E-175 |
|  | 393 | RED | Red | 1 | PONAB | 6.39E-160 |
|  | 768 | RPKL1 | Ribosomal S6 kinase-like 1 | 1 | PONAB | 1.05E-18 |
|  | 521 | S6A12 | Sodium- and chloride-dependent betaine transporter | 1 | MOUSE | 0 |
|  | 321 | S6A13 | Sodium- and chloride-dependent GABA transporter 2 | 5 | RAT | 5.10E-149 |
|  | 130 | S6A14 | Sodium- and chloride-dependent neutral and basic amino acid transporter B(0+) | 1 | HUMAN | 4.84E-16 |
|  | 289 | SC6A1 | Sodium- and chloride-dependent GABA transporter 1 | 2 | RAT | 1.11E-88 |
|  | 322 | SCD5 | Stearoyl- desaturase 5 | 1 | BOVIN | 0 |
|  | 322 | SCEL | Sciellin | 1 | HUMAN | 0 |
|  | 262 | SRF | Serum response factor | 1 | HUMAN | 2.05E-94 |
|  | 649 | SRFB1 | Serum response factor-binding 1 | 1 | MOUSE | 6.46E-84 |
|  | 546 | STK4 | Serine threonine- kinase 4 | 1 | BOVIN | 0 |
|  | 224 | TICN1 | Testican-1 | 1 | HUMAN | 4.38E-102 |
|  | 681 | TRPV2 | Transient receptor potential cation channel subfamily V member 2 | 3 | RAT | 1.04E-100 |
|  | 978 | TRPV4 | Transient receptor potential cation channel subfamily V member 4 | 1 | HUMAN | 1.34E-67 |
|  | 889 | TRPV5 | Transient receptor potential cation channel subfamily V member 5 | 1 | HUMAN | 0 |
|  | 274 | TYB11 | Thymosin beta-11 | 1 | ONCMY | 1.01E-144 |
|  | 533 | ULK1 | Serine threonine- kinase ULK1 | 1 | HUMAN | 0 |
|  | 383 | UT14A | U3 small nucleolar RNA-associated 14 homolog A | 1 | BOVIN | 3.80E-102 |
|  | 452 | UTP11 | Probable U3 small nucleolar RNA-associated 11 | 1 | HUMAN | 1.42E-158 |
|  | 582 | V2R | Vasopressin V2 receptor | 3 | MOUSE | 9.02E-81 |
|  | 196 | V2R1 | Vomeronasal type-2 receptor 1 | 3 | MOUSE | 4.12E-51 |
|  | 1395 | WFS1 | Wolframin | 1 | MOUSE | 0 |
|  | 908 | WHAMM | WASP homolog-associated with membranes and microtubules | 1 | HUMAN | 0 |
|  | 193 | YAED1 | Yae1 domain-containing 1 | 1 | HUMAN | 6.83E-17 |

**Table S22. 512 homeostasis Genes in different molecular functions in terms of functional KEGG Orthology**.

| **1st Hierarchy** | **2nd Hierarchy** | **No. of Genes identified** |
| --- | --- | --- |
| Metabolism | Carbohydrate metabolism | 10 |
|  | Lipid metabolism | 10 |
|  | Nucleotide metabolism | 7 |
|  | Amino acid metabolism | 6 |
|  | Metabolism of cofactors and vitamins | 5 |
|  | Glycan biosynthesis and metabolism | 4 |
|  | Energy metabolism | 2 |
|  | Metabolism of other amino acids | 1 |
| Genetic Information Processing | Folding, sorting and degradation | 8 |
|  | Translation | 2 |
| Environmental Information Processing | Signal transduction | 84 |
|  | Signaling molecules and interaction | 30 |
|  | Membrane transport | 6 |
| Cellular Processes | Cellular community - eukaryotes | 34 |
|  | Cell growth and death | 23 |
|  | Cell motility | 10 |
|  | Transport and catabolism | 10 |
| Organismal Systems | Digestive system | 54 |
|  | Endocrine system | 48 |
|  | Immune system | 38 |
|  | Nervous system | 37 |
|  | Circulatory system | 25 |
|  | Excretory system | 25 |
|  | Sensory system | 23 |
|  | Environmental adaptation | 16 |
|  | Aging | 13 |
|  | Development | 8 |

Table S23 Total 54 genes categorized under Pathways in Digestive systems under Organismal Systems through KEGG analysis

| **S.No.** | **3rd hierarchy** | **No. of Gene** |
| --- | --- | --- |
| 1 | Gastric acid secretion [PATH:ko04971] | 23 |
| 2 | Bile secretion [PATH:ko04976] | 17 |
| 3 | Pancreatic secretion [PATH:ko04972] | 15 |
| 4 | Mineral absorption [PATH:ko04978] | 12 |
| 5 | Protein digestion and absorption [PATH:ko04974] | 6 |
| 6 | Carbohydrate digestion and absorption [PATH:ko04973] | 3 |
| 7 | Cholesterol metabolism [PATH:ko04979] | 2 |

**Table S24. Total 48 genes categorized with GO related to homeostasis** under Pathways in Endocrine systems under Organismal Systems through KEGG analysis

| **S No** | **3rd hierarchy** | **No. of Gene** |
| --- | --- | --- |
| 1 | Parathyroid hormone synthesis, secretion and action [PATH:ko04928] | 23 |
| 2 | Oxytocin signaling pathway [PATH:ko04921] | 17 |
| 3 | Renin-angiotensin system [PATH:ko04614] | 16 |
| 4 | Insulin secretion [PATH:ko04911] | 15 |
| 5 | Thyroid hormone synthesis [PATH:ko04918] | 15 |
| 6 | Estrogen signaling pathway [PATH:ko04915] | 14 |
| 7 | GnRH signaling pathway [PATH:ko04912] | 14 |
| 8 | Renin secretion [PATH:ko04924] | 14 |
| 9 | Relaxin signaling pathway [PATH:ko04926] | 13 |
| 10 | Cortisol synthesis and secretion [PATH:ko04927] | 12 |
| 11 | Glucagon signaling pathway [PATH:ko04922] | 12 |
| 12 | Melanogenesis [PATH:ko04916] | 11 |
| 13 | Regulation of lipolysis in adipocytes [PATH:ko04923] | 11 |
| 14 | Ovarian steroidogenesis [PATH:ko04913] | 8 |
| 15 | Progesterone-mediated oocyte maturation [PATH:ko04914] | 8 |
| 16 | Thyroid hormone signaling pathway [PATH:ko04919] | 8 |
| 17 | Insulin signaling pathway [PATH:ko04910] | 7 |
| 18 | Adipocytokine signaling pathway [PATH:ko04920] | 3 |
| 19 | Prolactin signaling pathway [PATH:ko04917] | 2 |

Table S25 Total 26 genes with GO related to osmoregulation ,categorized under Pathways in excretory systems under Organismal Systems through KEGG analysis

| **3rd hierarchy** | **No. of Genes** |
| --- | --- |
| Aldosterone-regulated sodium reabsorption [PATH:ko04960] | 9 |
| Endocrine and other factor-regulated calcium reabsorption [PATH:ko04961] | 9 |
| Vasopressin-regulated water reabsorption [PATH:ko04962] | 9 |
| Collecting duct acid secretion [PATH:ko04966] | 4 |
| Proximal tubule bicarbonate reclamation [PATH:ko04964] | 2 |

**Table S26: Multiple copies of Osmo-regulatory related genes in *Tenualosa ilisha*** genome.

| SNo | **Claudins**  **(23 genes)** | | **Aquaporins**  **(9 genes)** | | **Connexins/Gap junctions**  **(22 genes)** | | **Adenylate cyclase**  **(8 genes)** | | **Solute carriers***  **(34 genes)** | | **Voltage gated potassium channel gene**  **(45 genes)** | |
| --- | --- | --- | --- | --- | --- | --- | --- | --- | --- | --- | --- | --- |
|  | **Gene** | **No of copies** | **Gene** | **No of copies** | **Gene** | **No of copies** | **Gene** | **No of copies** | **Gene** | **No of copies** | **Gene** | **No of copies** |
| 1 | *CLD3* | 2 | ***AQP1*** | ***5*** | ***CX32*** | ***6*** | ADCY1 | 1 | **SLC12A4_5_6** | 4 | KCA10 | 2 |
| 2 | ***CLD4*** | ***13*** | *AQP3* | 2 | *CX33* | 1 | ADCY2 | 4 | **SLC14A** | 2 | KCAB1 | 9 |
| 3 | *CLD5* | 3 | *AQP4* | 3 | *CXA1* | 2 | ADCY3 | 2 | **SLC24A2** | 1 | KCAB2 | 2 |
| 4 | *CLD6* | 3 | *AQP7* | 1 | *CXA3* | 4 | ADCY5 | 2 | **SLC24A3** | 1 | KCAB3 | 1 |
| 5 | *CLD7* | 2 | *AQP8* | 3 | *CXA4* | 1 | ADCY6 | 1 | **SLC24A4** | 2 | KCNA1 | 2 |
| 6 | *CLD8* | 5 | *AQP9* | 3 | *CXA5* | 3 | ADCY8 | 9 | **SLC24A6** | 1 | KCNA2 | 5 |
| 7 | *CLD10* | 5 | *AQP10* | 1 | *CXA8* | 3 | ADCY9 | 2 | SLC25A23S | 4 | KCNA3 | 2 |
| 8 | *CLD11* | 2 | *AQP11* | 2 | *CXA9* | 3 | ADCYA | 1 | SLC30A1 | 3 | KCNA4 | 2 |
| 9 | *CLD12* | 1 | *AQP12* | 1 | *CXA10* | 2 | **TOTAL** | **22** | SLC31A1 | 1 | KCNA5 | 1 |
| 10 | *CLD14* | 2 | **TOTAL** | **21** | *CXB1* | 3 |  |  | **SLC34A** | 3 | KCNB1 | 4 |
| 11 | *CLD15* | 1 |  |  | *CXB3* | 2 |  |  | SLC35B4 | 1 | KCNB2 | 3 |
| 12 | *CLD18* | 1 |  |  | *CXB4* | 5 |  |  | SLC39A1_2_3 | 3 | KCNB2 | 1 |
| 13 | *CLD19* | 1 |  |  | *CXB6* | 2 |  |  | SLC39A10 | 1 | KCNC1 | 9 |
| 14 | *CLD20* | 2 |  |  | *CXB7* | 1 |  |  | SLC39A12 | 1 | KCNC2 | 2 |
| 15 | *CLD22* | 3 |  |  | *CXD2* | 5 |  |  | SLC39A13 | 2 | KCNC4 | 3 |
| 16 | *CLD23* | 3 |  |  | *CXD3* | 2 |  |  | SLC39A14 | 1 | KCND1 | 2 |
| 17 | *CLD34* | 2 |  |  | *CXD4* | 1 |  |  | SLC39A6 | 1 | KCND2 | 5 |
| 18 | *TM204* | 1 |  |  | *CXE1* | 1 |  |  | SLC39A7 | 1 | KCND3 | 4 |
| 19 | *TM235* | 2 |  |  | ***CXG1*** | ***6*** |  |  | SLC39A8 | 1 | KCNE1 | 1 |
| 20 | *CLD7A* | 4 |  |  | *RIC1* | 1 |  |  | SLC40A1 | 2 | KCNE2 | 1 |
| 21 | *CLDN1* | 2 |  |  | *UBQL4* | 1 |  |  | **SLC41A** | 3 | KCNE4 | 2 |
| 22 | *CLDY* | 6 |  |  | **TOTAL** | **55** |  |  | **SLC4A1** | 1 | KCNF1 | 2 |
| 23 | *CLDZ* | 2 |  |  |  |  |  |  | **SLC4A11** | 1 | KCNG1 | 1 |
| 24 | **TOTAL** | **68** |  |  |  |  |  |  | SLC6A1 | 2 | KCNG2 | 2 |
| 25 |  |  |  |  |  |  |  |  | SLC6A11 | 2 | KCNG3 | 2 |
| 26 |  |  |  |  |  |  |  |  | SLC6A3 | 1 | KCNG4 | 2 |
| 27 |  |  |  |  |  |  |  |  | SLC6A6 | 1 | KCNH1 | 4 |
| 28 |  |  |  |  |  |  |  |  | SLC7A10 | 2 | KCNH2 | 4 |
| 29 |  |  |  |  |  |  |  |  | SLC7A5 | 1 | KCNH4 | 3 |
| 30 |  |  |  |  |  |  |  |  | SLC7A6 | 1 | KCNH5 | 5 |
| 31 |  |  |  |  |  |  |  |  | SLC7A8 | 2 | KCNH6 | 3 |
| 32 |  |  |  |  |  |  |  |  | **SLC8A** | 4 | KCNH7 | 6 |
| 33 |  |  |  |  |  |  |  |  | **SLC9A1** | 3 | KCNH8 | 3 |
| 34 |  |  |  |  |  |  |  |  | **SLC9A2** | 1 | KCNQ1 | 5 |
| 35 |  |  |  |  |  |  |  |  | **TOTAL** | 61 | KCNQ2 | 5 |
| 36 |  |  |  |  |  |  |  |  |  |  | KCNQ3 | 4 |
| 37 |  |  |  |  |  |  |  |  |  |  | KCNQ4 | 3 |
| 38 |  |  |  |  |  |  |  |  |  |  | KCNQ5 | 6 |
| 39 |  |  |  |  |  |  |  |  |  |  | KCNS1 | 1 |
| 40 |  |  |  |  |  |  |  |  |  |  | KCNS2 | 1 |
| 41 |  |  |  |  |  |  |  |  |  |  | KCNS3 | 2 |
| 42 |  |  |  |  |  |  |  |  |  |  | KCNT1 | 1 |
| 43 |  |  |  |  |  |  |  |  |  |  | KCNT2 | 2 |
| 44 |  |  |  |  |  |  |  |  |  |  | KCNV1 | 1 |
| 45 |  |  |  |  |  |  |  |  |  |  | KCNV2 | 4 |
|  |  |  |  |  |  |  |  |  |  |  |  | 135 |

*Bold: families involved in Inorganic cation/anion transport

.

**Table S27** Summary of Orthology analysis done by OrthoFinder program

| **Orthology Description** | **Statistics** |
| --- | --- |
| Number of genes | 687245 |
| Number of genes in orthogroups | 583153 |
| Number of unassigned genes | 104092 |
| Percentage of genes in orthogroups | 84.9% |
| Percentage of unassigned genes | 15.1% |
| Number of orthogroups | 28249 |
| Number of species-specific orthogroups | 518 |
| Number of genes in species-specific orthogroups | 3175 |
| Percentage of genes in species-specific orthogroups | 0.5 |
| Mean orthogroup size | 20.6 |
| Median orthogroup size | 14 |
| G50 (assigned genes) | 31 |
| G50 (all genes) | 25 |
| O50 (assigned genes) | 4489 |
| O50 (all genes) | 6365 |
| Number of orthogroups with all species present | 39 |

**Table S28 Summary of Orthology analysis of 12 species by OrthoFinder program, where 55 genes of *Tenualosa ilisha* in species-specific orthogroups**

| **Orthology Description/Species** | ***Clupea harengus*** | ***Cyprinus carpio*** | ***Danio rerio*** | ***Dicentrarchus labrax*** | ***Esox lucius*** | ***Gasterosteus aculeatus*** | ***Late calcarifer*** | ***Mororn saxatilis*** | ***Oncorhynchus mykiss*** | ***Oreochromis niloticus*** | ***Salmo salar*** | ***Tenualosa ilisha*** |
| --- | --- | --- | --- | --- | --- | --- | --- | --- | --- | --- | --- | --- |
| Number of genes | 29855 | 64455 | 54387 | 15082 | 65858 | 4578 | 45752 | 14555 | 144253 | 97968 | 116800 | 33696 |
| Number of genes in orthogroups | 29262 | 57931 | 52308 | 12192 | 64024 | 3946 | 44885 | 13082 | 95533 | 85583 | 93426 | 30981 |
| Number of unassigned genes | 593 | 6524 | 2079 | 2890 | 1834 | 632 | 867 | 1473 | 48720 | 12385 | 23374 | 2694 |
| Percentage of genes in orthogroups | 98 | 89.9 | 96.2 | 80.8 | 97.2 | 86.2 | 98.1 | 89.9 | 66.2 | 87.4 | 80 | 91.7 |
| Percentage of unassigned genes | 2 | 10.1 | 3.8 | 19.2 | 2.8 | 13.8 | 1.9 | 10.1 | 33.8 | 12.6 | 20 | 8.3 |
| Number of orthogroups containing target species | 15464 | 18463 | 16527 | 4623 | 16866 | 268 | 16180 | 1798 | 23184 | 19873 | 19497 | 17015 |
| Percentage of orthogroups containing targetspecies | 54.7 | 65.4 | 58.5 | 16.4 | 59.7 | 0.9 | 57.3 | 6.4 | 82.1 | 70.3 | 69 | 60.2 |
| Number of species-specific orthogroups | 13 | 60 | 26 | 32 | 35 | 0 | 33 | 19 | 66 | 126 | 90 | 18 |
| Number of genes in species-specific orthogroups | 64 | 181 | 212 | 190 | 157 | 0 | 160 | 154 | 458 | 1019 | 525 | **55** |
| Percentage of genes in species-specific orthogroups | 0.2 | 0.3 | 0.4 | 1.3 | 0.2 | 0 | 0.3 | 1.1 | 0.3 | 1 | 0.4 | 0.2 |

**Table S29. *Tenualosa ilisha* species specific gene variants** identified through orthology analysis

| **Seq Name** | **Gene ID** | **Description** | **e-va**l**ues** | **KO term** | **GO_Name** | **GO_ID** |
| --- | --- | --- | --- | --- | --- | --- |
| g56609.t1 | CASR | Extracellular calcium-sensing receptor | 1.79E-30 | K04612 | C:cytoplasm;  F:ion binding; F:signal transducer activity; P:transport; P:circulatory system process; C:plasma membrane | C:GO:0005737; F:GO:0043167; F:GO:0004871;  P:GO:0006810; P:GO:0003013;  C:GO:0005886 |
| g56593.t1 | CASS4 | Cas scaffolding family member 4 | 7.7E-45 | K04612 | C:cytoplasm; F:ion binding; F:signal transducer activity; P:transport; P:circulatory system process; C:plasma membrane | C:GO:0005737; F:GO:0043167; F:GO:0004871; P:GO:0006810; P:GO:0003013; C:GO:0005886 |
| g34017.t1 | CNKR2 | Connector enhancer of kinase suppressor of ras 2 | 2.24E-09 | K17536 | P:biological_process; C:cytoplasm; C:organelle; C:plasma membrane; F:molecular_function | P:GO:0008150; C:GO:0005737; C:GO:0043226; C:GO:0005886; F:GO:0003674 |
| g45506.t1 | FIG4 | Polyphosphoinositide phosphatase | 1.13E-34 | K22913 | C:endosome; P:pigmentation; P:cellular component assembly; P:lipid metabolic process; F:phosphatase activity; P:cell differentiation; P:anatomical structure formation involved in morphogenesis; C:Golgi apparatus; C:endoplasmic reticulum; P:biosynthetic process; P:single-organism biosynthetic process; P:neurological system process | C:GO:0005768; P:GO:0043473; P:GO:0022607; P:GO:0006629; F:GO:0016791; P:GO:0030154; P:GO:0048646; C:GO:0005794; C:GO:0005783; P:GO:0009058; P:GO:0044711; P:GO:0050877 |
| g32379.t1 | KV13 | Ig kappa chain V region 3547 | 5.83E-29 | - | C:extracellular space; P:vesicle-mediated transport; P:signal transduction; F:peptidase activity; C:plasma membrane; P:immune system process | C:GO:0005615; P:GO:0016192; P:GO:0007165; F:GO:0008233; C:GO:0005886; P:GO:0002376 |
| g43563.t1 | LYAM1 | L-selectin, CD62 antigen-like family member L , Leukocyte adhesion molecule 1 | 7.54E-18 | K12316 | P:generation of precursor metabolites and energy; P:catabolic process; P:anatomical structure development; C:extracellular region; P:circulatory system process; C:plasma membrane; C:lysosome; P:single-organism carbohydrate metabolic process; P:single-organism catabolic process; P:small molecule metabolic process; P:carbohydrate metabolic process; P:homeostatic process; P:neurological system process; F:hydrolase activity, acting on glycosyl bonds | P:GO:0006091; P:GO:0009056; P:GO:0048856; C:GO:0005576; P:GO:0003013; C:GO:0005886; C:GO:0005764; P:GO:0044723; P:GO:0044712; P:GO:0044281; P:GO:0005975; P:GO:0042592; P:GO:0050877; F:GO:0016798 |
| g25341.t1 | MOG | Myelin-oligodendrocyte glycoprotein | 9.54E-10 | - | P:single-organism transport; P:transport; C:plasma membrane; P:immune system process | P:GO:0044765; P:GO:0006810; C:GO:0005886; P:GO:0002376 |
| g39495.t1 | NKAI3 | Sodium potassium-transporting ATPase subunit beta-1-interacting 3 | 6.71E-^25^ | BR:ko02000 | P:transport; C:plasma membrane | P:GO:0006810; C:GO:0005886 |
| g38335.t1 | PLXND1 | Plexin-D1 | 4.18E-06 | - | P:anatomical structure development | P:GO:0048856 |

**Table S30** Alignment Summary from comparative Synteny analysis of *Clupea harengus* and *Tenualosa ilisha* draft genomes.

Genome Statistics

| Species | #Seqs | Total kb | Max kb | Minkb | <100 kb | 100kb-1Mb | 1Mb-10Mb | >10Mb |
| --- | --- | --- | --- | --- | --- | --- | --- | --- |
| Clupea | 19 | 126456 | 13053 | 5080 | 0 | 0 | 20 | 1 |
| Hilsa | 23 | 170743 | 17426 | 5019 | 0 | 0 | 0 | 3 |

Anchor Statistics

| Species | Anchors | %InBlocks | %Coverage | <100bp | 100bp-1 kb | 1kb-10kb | >10kb |
| --- | --- | --- | --- | --- | --- | --- | --- |
| Clupea | 17183 | 58 | 19 | 234 | 10980 | 5766 | 203 |
| Hilsa | 17183 | 58 | 14 | 283 | 11055 | 5635 | 210 |

Block Statistics

| Species | Blocks | %Coverage | %DoubleCov | Inverted | <100kb | 100kb-1Mb | 1Mb-10Mb | >10Mb |
| --- | --- | --- | --- | --- | --- | --- | --- | --- |
| Clupea | 51 | 50 | 4 | 22 | 5 | 27 | 19 | 0 |
| Hilsa | 51 | 36 | 0 | 22 | 9 | 21 | 21 | 0 |

***Table S32 Summary of mitochondrial genes of Tenualosa*** ilisha

| **S.No** | **Codon Start** | **Codon End** | **Gene** | **Product** |
| --- | --- | --- | --- | --- |
| 1 | 2851 | 3825 | ND1 | NADH dehydrogenase subunit 1 |
| 2 | 4041 | 5088 | ND2 | NADH dehydrogenase subunit 2 |
| 3 | 5476 | 7026 | COI | cytochrome c oxidase subunit I |
| 4 | 7183 | 7873 | COII | cytochrome c oxidase subunit II |
| 5 | 7949 | 8116 | ATPase 8 | ATPase subunit 8 |
| 6 | 8107 | 8789 | ATPase 6 | ATPase subunit 6 |
| 7 | 8790 | 9574 | COIII | cytochrome c oxidase subunit III |
| 8 | 9646 | 9994 | ND3 | NADH dehydrogenase subunit 3 |
| 9 | 10064 | 10360 | ND4L | NADH dehydrogenase subunit 4L |
| 10 | 10354 | 11734 | ND4 | NADH dehydrogenase subunit 4 |
| 11 | 11943 | 13790 | ND5 | NADH dehydrogenase subunit 5 |
| 12 | 13775 | 14296 | ND6 | NADH dehydrogenase subunit 6 |
| 13 | 14371 | 15511 | Cyt b | cytochrome b |
| **Ribosomal RNA genes** | | | | |
| 1 | 70 | 1023 | rRNA | 12S rRNA |
| 2 | 1096 | 2775 | rRNA | 16S rRNA |
| **tRNA genes** | | | | |
| 1 | 1 | 69 | tRNA | tRNA-Phe |
| 2 | 1024 | 1095 | tRNA | tRNA-Val |
| 3 | 2776 | 2850 | tRNA | tRNA-Leu |
| 4 | 3831 | 3902 | tRNA | tRNA-Ile |
| 5 | 3902 | 3972 | tRNA | tRNA-Gln |
| 6 | 3972 | 4040 | tRNA | tRNA-Met |
| 7 | 5089 | 5160 | tRNA | tRNA-Trp |
| 8 | 5163 | 5231 | tRNA | tRNA-Ala |
| 9 | 5233 | 5305 | tRNA | tRNA-Asn |
| 10 | 5338 | 5403 | tRNA | tRNA-Cys |
| 11 | 5405 | 5474 | tRNA | tRNA-Tyr |
| 12 | 7027 | 7097 | tRNA | tRNA-Ser |
| 13 | 7102 | 7170 | tRNA | tRNA-Asp |
| 14 | 7874 | 7947 | tRNA | tRNA-Lys |
| 15 | 9575 | 9645 | tRNA | tRNA-Gly |
| 16 | 9995 | 10063 | tRNA | tRNA-Arg |
| 17 | 11735 | 11803 | tRNA | tRNA-His |
| 18 | 11804 | 11870 | tRNA | tRNA-Ser |
| 19 | 11871 | 11942 | tRNA | tRNA-Leu |
| 20 | 14297 | 14365 | tRNA | tRNA-Glu |
| 21 | 15512 | 15583 | tRNA | tRNA-Thr |
| 22 | 15583 | 15652 | tRNA | tRNA-Pro |
| **Other genes** | | | | |
| 1 | 15653 | 16745 | D-loop | control region |
